# Advances in the study of IL-17 in neurological diseases and mental disorders

**DOI:** 10.3389/fneur.2023.1284304

**Published:** 2023-11-16

**Authors:** Yu Lu, Piaopiao Zhang, Fenfen Xu, Yuan Zheng, Hongyang Zhao

**Affiliations:** ^1^Department of Pediatrics, Jinan Central Hospital, Shandong University, Jinan, China; ^2^Department of Pediatrics, Central Hospital Affiliated to Shandong First Medical University, Jinan, China

**Keywords:** IL-17, neurological diseases, mental disorders, neuroinflammation, microbiota-gut-brain axis

## Abstract

Interleukin-17 (IL-17), a cytokine characteristically secreted by T helper 17 (Th17) cells, has attracted increasing attention in recent years because of its importance in the pathogenesis of many autoimmune or chronic inflammatory diseases. Recent studies have shown that neurological diseases and mental disorders are closely related to immune function, and varying degrees of immune dysregulation may disrupt normal expression of immune molecules at critical stages of neural development. Starting from relevant mechanisms affecting immune regulation, this article reviews the research progress of IL-17 in a selected group of neurological diseases and mental disorders (autism spectrum disorder, Alzheimer’s disease, epilepsy, and depression) from the perspective of neuroinflammation and the microbiota–gut–brain axis, summarizes the commonalities, and provides a prospective outlook of target application in disease treatment.

## 1 Introduction

Interleukin-17A (IL-17A), also known as IL-17, is a signature cytokine secreted by T helper 17 (Th17) cells, which play an essential role in maintaining immune homeostasis and promoting immune dysregulation. Currently, IL-17 has been studied in immune diseases, tumor diseases, and chronic inflammatory diseases, such as multiple sclerosis (MS), rheumatoid arthritis, ankylosing spondylitis, and psoriasis (1). Contemporary research has also suggested that the pathogenesis of many neurological diseases or mental disorders is closely related to autoimmune or inflammatory injuries and that varying degrees of immune dysregulation are prevalent both centrally and peripherally, which may disrupt the normal expression of immune molecules at the critical stages of neurodevelopment (2).

Neurological disorders are increasingly recognized as the leading cause of death and disability worldwide; moreover, mental disorders are not to be overlooked either, with depression accounting for the highest proportion of diagnoses (3). In recent years, it has been noted that neuroinflammation and gut microecology can mediate and regulate immunity in both central nervous system (CNS) diseases and mental disorders. Neuroinflammation usually refers to the inflammatory response within the CNS and can be caused by infection, trauma, ischemia, and other pathological injuries. This process is characterized by the production of proinflammatory cytokines that mediate neuronal death, neurogenesis inhibition, and synaptic dysfunction, whereas glial cells may act as the primary immune cells for a rapid immune response (4, 5). The gut is no longer considered solely as a digestive organ; its interactions with the immune system are equally complex. The intestinal barrier is in close contact with the intestinal microbiota on its luminal side and with enteric neurons and neuroglia on its tissue side and is closely associated with disorders of the CNS (6). For this review, we selected a group of neurological diseases and mental disorders, including autism spectrum disorder (ASD), Alzheimer’s disease (AD), epilepsy, and depression. By reading relevant reviews and summarizing a large number of basic and clinical studies, it was found that, although the current studies on IL-17 have been more extensively and comprehensively elucidated in several neurological diseases and mental disorders, unlike a previous study on IL-17 that has been mostly limited to mechanisms such as neuroinflammatory effects, our review additionally adopts the microbiota–gut–brain axis as a novel perspective to discuss how IL-17 directly or indirectly influences the immune system in physiological and pathological contexts. Within the selected group of neurological diseases and mental disorders, we did not simply analyze the unilinear association between IL-17 and each disease but rather interactively analyzed the neuroinflammation and microbiota–gut–brain axis pathways of the four diseases, uncovering the commonalities of IL-17 action in them. Linking the four diseases through IL-17-related mechanisms makes the review more comprehensive and is crucial for finding more effective disease intervention targets.

## 2 Overview of IL-17

### 2.1 Biological properties of IL-17

IL-17A, or IL-17, initially named cytotoxic T lymphocyte-associated antigen 8 (CTLA8) and first cloned in 1993 from a rodent ablation cross (7), is the signature cytokine produced by Th17 cells. IL-17A is the most well-studied effector within the IL-17 cytokines family, sharing approximately 55% sequence homology with IL-17F, which is co-expressed in a linked gene. Other structurally related members in the family include IL-17B, IL-17C, IL-17D, and IL-17E (IL-25) (8). IL-17 is a dimeric molecule consisting of 163–202 amino acids linked by disulfide bonds with a molecular weight ranging from 23 to 36 kDa. It exhibits a cysteine knot-monomer folding structure comparable to that of nerve growth factor (NGF) and platelet-derived growth factor (PDGF). Despite this structural similarity, IL-17 shows no homology with other cytokines due to the presence of unstable adenylate-uridylate-rich sequences in its 3′ untranslated region (9). The IL-17-binding receptor (IL-17R) can be categorized into five types, i.e., IL-17R (A-E). IL-17RA, a co-receptor common to the IL-17 family of ligands, is the most critical among them (10).

IL-17 is mainly derived from Th17 cells and is the main effector cytokine of Th17 cells. However, studies indicated that other lymphocytes involved in innate or adaptive immunity can also secrete IL-17, such as γδ-T cells, αβ-T cells, invariant natural killer T (iNKT) cells, CD8+ T cells [cytotoxic T cells 17 (Tc17)], and type 3 innate lymphocytes (ILC3). Additionally, astrocytes and microglia of the CNS also express IL-17 (11). Corresponding to the ligand, IL-17R is widely distributed in a variety of tissues as well as within the CNS; the monomers IL-17A and IL-17F signal through the formation of homodimeric and heterodimeric IL-17A and IL-17F, respectively, which binds to the specialized dimeric receptor complexes IL-17RA and IL-17RC (12).

The role of IL-17 has been better understood over the years due to its frequent appearance in a variety of diseases, but its existence can be both a blessing and a curse in immunomodulation. On the one hand, it can act as protective immunity by rapidly mediating the chemokine recruitment of neutrophil and promoting antimicrobial peptide (AMP) production in acute inflammation, as well as promoting cell proliferation and barrier tissue repair to protect the host from mucosal infection (13). On the other hand, when IL-17 is dysfunctional, it not only promotes infection and autoimmunity due to the stimulus of chronic inflammation but also leads to long-term pathological repair, which has been linked to the development of malignant tumors (14). Abnormal expression of IL-17 can be observed in several neurological or mental diseases in the present research, and this expression is closely related to the immunomodulation it induces.

### 2.2 IL-17 signaling pathway

#### 2.2.1 IL-17 secretion pathway

In 2005, Park et al. first defined cells characteristically secreting IL-17 as Th17 cells, a novel subtype of CD4+ T lymphocytes distinct from Th1 and Th2 lineage cells characteristically expressing interferon-gamma (IFN-γ) and IL-4 (15). All Th17 cells carry the C-C chemokine receptor 6 (CCR6) and express the retinoid-related orphan receptor gamma t (RORγt), which is considered a key transcription factor for Th17 cell differentiation (16). The research established that transforming growth factor β (TGF-β) and IL-6 initiate initial naive CD4+ T differentiation which is then enhanced and stabilized by IL-23; eventually, signal transducer and activator of transcription 3 (STAT3) and the transcription factor RORγt mediate the differentiation of Th17 lineage cells. Within this process, IL-23 must be present in combination with other cytokines to function as its receptor is not expressed in the initial T cells (17). In addition to IL-17A, Th17 cells produce IL-17F, IL-21, IL-22, granulocyte-macrophage colony-stimulating factor (GM-CSF), IFN-γ, tumor necrosis factor (TNF), and IL-26 in humans (18). Among them, host protective Th17 cells expressed both IL-17 and IL-10, while the highly inflammatory Th17 cell population expressed IL-17, IL-22, and GM-CSF (17). Regulatory T (Treg) cells play the opposite role to Th17 cells in the immune response; they mediate immune tolerance and act as the key to suppressing the excessive inflammatory activity of Th17 cells (19). Treg cell differentiation is driven by the transcription factor forkhead box P3 (FoxP3), which is reduced in individuals with inflammatory neurological diseases, and reduced Treg cell function is associated with the upregulation of RORγt (20). Studies of newly defined STAT3-dependent Th17-specific regulatory T (Treg17) cells have revealed that selective deletion of murine STAT3 leads to Treg17 cell deficiency, which in turn leads to an excessive Th17 cell response (21). Moreover, co-expression of CCR6 is a mechanism by which Treg17 cells are able to target pathogenic Th17 cell responses (21). The imbalance between Th17 and Treg cells is crucial in autoimmunity, but its role in neurological or mental disorders has not been fully elucidated.

#### 2.2.2 IL-17 pathway of action

**Figure 1:**
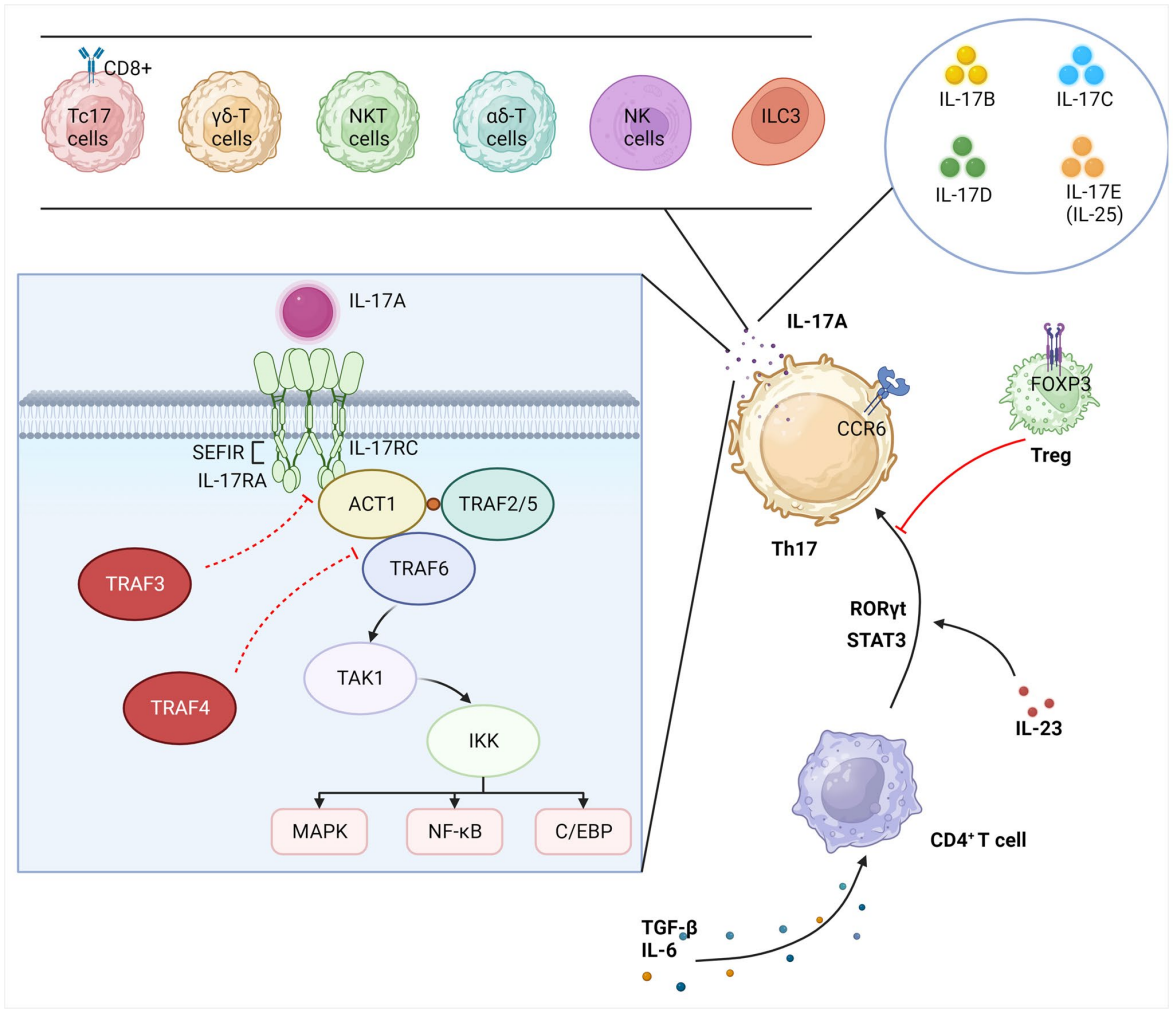
Overview of IL-17. IL-17A is secreted primarily by Th17 cells specifically but can also be secreted by other immune cells. The IL-17 family also includes IL-17(B-E). CD4+ T cells differentiate Th17 lineage cells in response to several inflammatory and transcription factors. Treg cells act as the key to suppressing the excessive inflammatory activity of Th17 cells. IL-17A triggers the transcription of IL-17 target genes by binding to the IL-17 receptor complex, which activates three major pathways, namely, NF-κB, MAPK, and C/EBP. This signaling cascade is regulated in multiple steps. Th17, T helper 17; Treg, regulatory T; iNKT, invariant natural killer T; Tc17, cytotoxic T cells 17; ILC3, type 3 innate lymphocytes; CCR6, C-C chemokine receptor 6; RORγt, retinoid-related orphan receptor gamma t; STAT3, signal transducer and activator of transcription 3; FoxP3, forkhead box P3; IL, interleukin; TGF-β, transforming growth factor β; IL-17R, IL-17-binding receptor; TRAF, TNF receptor-associated factor; ACT1, NF-κB activator 1; TAK1, transforming growth factor-β-activated kinase-1; IKK, inhibitor of NF-κB kinase; SEFIR, similar expression of fibroblast growth factor; MAPK, mitogen-activated protein kinase; NF-κB, nuclear factors κB; C/EBP, CCAA T/enhancer-binding protein.

After binding to the receptor, IL-17 family members are bound by IL-17RA and the junction protein ACT1 (NF-κB activator 1) through a conserved motif called the “Similar expression of fibroblast growth factor genes and IL-17Rs” (SEFIR) structural domain. This domain is expressed similarly to IL-17R and appears to be associated with IL-1 and “TIR” (Toll/IL-1R) structural domains (22). A number of regulators have been identified to be of significance in this signaling cascade, such as the bridging protein TNF receptor-associated factor 3 (TRAF3), which inhibits the binding of IL-17RA to Act1, and TRAF4, which interferes with the interaction between Act1 and TRAF6 (11). Recently, Miyashita et al. discovered that TIR-containing adapter molecule 1 (TICAM-1) which belongs to TIR, a structural domain similar to SEFIR, inhibits the interaction between Act1 and IL-17RA and attenuates IL-17A-mediated cytokine and chemokine expression (23). ACT1 and TRAF6, both lysine 63 (K63) E3 ubiquitin ligases, are key links in the activation of the NF-κB pathway. TRAF6 is recruited and ubiquitinated by ACT1, which activates transforming growth factor-β-activated kinase-1 (TAK1) and the inhibitor of NF-κB kinase (IKK) complex, promoting the transcription and translation of related genes and cytokine production, such as IL-17, by phosphorylating the inhibitor of NF-κB (IκB) and exposing NF-κB nuclear localization signals (NLS). TRAF2 and TRAF5 may be involved downstream of inducible IκB kinase (IKKi) in mRNA stability driven by IL-17A (9). In addition to its role in cellular inflammation and immune response, NF-κB has been implicated in synaptic plasticity and learning and memory (24). In addition to the NF-κB pathway, IL-17 is also involved with the mitogen-activated protein kinase (MAPK) pathway. This pathway involves extracellular signal-regulated kinase (ERK), p38, and JUN N-terminal kinase (JNK) and is correlated with negative regulation mediated by CCAA T/enhancer-binding protein (C/EBP) (25). The entire section 2 is summarized in Figure 1.

## 3 IL-17 mediates immunomodulation-related pathways

### 3.1 IL-17 and neuroinflammation

IL-17 has been revealed to have a strong association with neuroinflammation. Neuroinflammation can disrupt the blood–brain barrier (BBB), cause abnormalities in glial and neuronal morphology and distribution, and impair synaptic plasticity, resulting in neurological or mental disease (26).

Microglia are the primary immune and cytokine-producing cells of the CNS and are essential for normal brain development. IL-17 can exacerbate neuroinflammation and neuropathology by activating microglia in animal models of Parkinson’s disease (27). Furthermore, it can compromise the integrity of the BBB and be implicated in lipopolysaccharide (LPS)-induced neuroinflammation and cognitive deficits in aged rats by activating microglia, whereas application of IL-17 antibodies (Abs) inhibits microglial activation and improves cognitive function (28). Another study established that IL-17 was released from activated microglia and promoted neuronal apoptosis after oxygen–glucose deprivation/reperfusion (OGDR) in rats (29). Astrocytes actively contribute to the formation and maintenance of the BBB and are substantial in preventing neurotoxic plasma components, blood cells, and pathogens from entering the brain. In experimental autoimmune encephalomyelitis (EAE), an animal model of MS, knockdown of Act1 expression in astrocytes blocks the IL-17 pathway (30). Investigations on the pathway of IL-17 to downstream signaling revealed that nuclear translocation of NF-κB in astrocytes can be triggered by IL-17 (31). Lin et al. concluded that, in astrocytes, IL-17 expressed through the p38/MAPK signaling pathway is critical for the differentiation of neural progenitor cells into neurons (32). Furthermore, IL-17 can inhibit astrocytes’ ability to convert glutamate into non-toxic glutamine, promoting glutamate excitotoxicity and mediating neurodegenerative lesions (33).

IL-17 has a dose-dependent effect on promoting neuronal damage in an *in vitro* brain injury model of OGD, except in glial cells (34). It is noteworthy that IL-17 can also affect the proliferation and differentiation of neural stem cells and neural progenitor cells involved in CNS neurogenesis. For example, sleep deprivation may interfere with the proliferation of mature neuronal precursor cells in the hippocampus through the IL-17 pathway, which may affect the cognitive level and emotion regulation (35). Furthermore, IL-17 has been shown to reduce the proliferation of hippocampal neural progenitor cells in the early stages of neurogenesis and promote neuronal differentiation and maturation in the later stages (36). Nevertheless, the molecular mechanisms underlying the effects of IL-17 on neurogenesis, neurons, glial cells, and the BBB in neurologic diseases or mental disorders have not been fully decoded and need to be further explored.

### 3.2 IL-17 and the microbiota–gut–brain axis

Over the past few years, there has been a growing recognition of the significant role that communication between the gut and the brain plays in maintaining human health. Through mechanisms such as immune activation, production of microbial metabolites, and various neurotransmitters in the gut itself, two-way communication between the gut flora and the CNS can influence neurodevelopment and social behavior in different species; this two-way communication is also known as the microbiota–gut–brain axis (37).

IL-17 has been shown to transduce inflammatory signals through the microbiota–gut–brain axis. Gut microbiota (GM) colonizes the gastrointestinal tract and consists of a diverse microbial community including bacteria, fungi, viruses, and protozoa, both beneficial and pathogenic, that can effectively regulate IL-17 and exert dual physiological and pathological effects on the host by upregulating IL-17 (13, 38). Restoration of intestinal Th17 cells and protection of intestinal homeostasis in an IL-17-dependent manner can be induced by the addition of segmented filamentous bacteria (SFB), under a high-fat, high-sugar diet (39). In contrast, elevated SFB also induces IL-17 expression in a pathological manner, and pregnant rats colonized with SFB or human commensal bacteria that induce gut Th17 cells have a higher likelihood of producing offspring with behavioral dysfunction resembling changes observed in ASD, which increases the risk of neurodevelopmental defects (40). Regen et al. revealed that IL-17-deficient mice had reduced susceptibility to EAE, but this susceptibility was renormalized after the restoration of homeostasis of intestinal microecology or IL-17 expression in the intestinal epithelium (41). Studies in mice lacking IL-17R, particularly on intestinal epithelial cells, demonstrated a delicate balance between IL-17 and the microbiota: IL-17 routinely regulates bacterial growth, potentially leading to intestinal ecological dysregulation, which in turn drives the activation of Th17 cells, resulting in increased IL-17 secretion (42). An imbalance of gut bacterial homeostasis promotes γδ-T cell migration to the meninges and production of IL-17 to exacerbate ischemic brain injury by altering dendritic cell activity (43).

In addition to colony-activated immunomodulation, microbial metabolite-dependent mechanisms are also implicated in the modulation of the CNS. Findings suggest that regulation of glycolytic pathways and lipid metabolism can influence Th17 cell differentiation (44). Hang et al. reported that the bile acid metabolite 3-oxoLCA inhibited Th17 cell differentiation and reduced IL-17 secretion by directly binding to the key transcription factor RORγt, whereas isoalloLCA promoted Treg cell differentiation by inducing mitochondrial reactive oxygen species production (45). A study by Hammer et al. showed that high dietary salt or increased intake of long-chain fatty acids (LCFAs) mediated increased infiltration of Th17 cells into the CNS, exacerbating neuroinflammation (46). Furthermore, short-chain fatty acids (SCFAs) reduce IL-17 expression in the gut and improve cognition (47).

**Figure 2:**
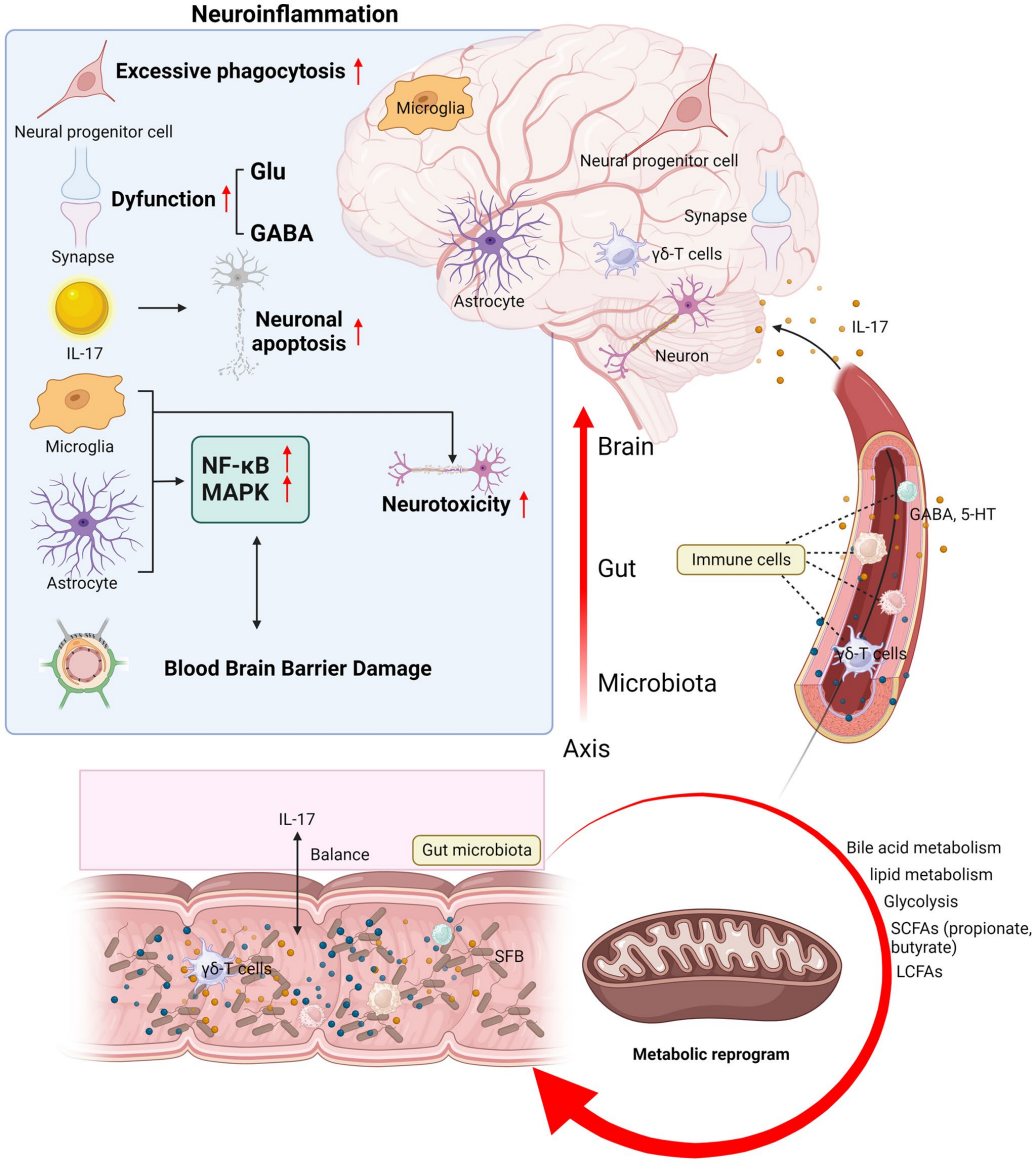
Mechanisms of IL-17 mediating immunoregulation in neurological diseases and mental disorders. IL-17 mediates the mechanisms of neuroinflammation and microbiota–gut–brain axis in neurological diseases and mental disorders. The brain section shows the mechanism of IL-17 and neuroinflammation: IL-17 disrupts the BBB, induces neuronal injury and apoptosis, regulates neurotoxicity and signal transduction in glial cells, affects neural progenitor cell proliferation and differentiation, and leads to dysfunctional synaptic transmission. The section on microbiota–gut–brain axis shows the balance between IL-17 and the gut microbiota, the migration of immune cells such as γδ-T cells from the gut to the meninges, and how the gut metabolites and neurotransmitters are regulated by IL-17 and then transported to the central nervous system for action. Glu, glutamate; GABA, gamma-aminobutyric acid; 5-HT, 5-hydroxytryptamine; SCFAs, short-chain fatty acids; LCFAs, long-chain fatty acids; SFB, segmented filamentous bacteria; BBB, blood–brain barrier.

Moreover, some neurotransmitters in the microbiota–gut–brain pathway can interact with IL-17, such as gamma-aminobutyric acid (GABA) and serotonin (5-hydroxytryptamine, 5-HT). GABA produced by *Lactococcus lactis* promotes IL-17 expression in the intestine during infection (48). GABA is the most abundant inhibitory neurotransmitter and is widely distributed in the mammalian brain. It is expressed in interneurons that regulate local circuits and plays an important role in synaptic transmission, which is of great importance to many common mental disorders (49). Although there is increasing evidence that IL-17 figures prominently in neurological and mental disorders through the microbiota–gut–brain axis, the precise correlations remain elusive and various issues still require further exploration. The entire section 3 is summarized in Figure 2.

## 4 Role of IL-17 in neurological and mental diseases

### 4.1 IL-17 and autism spectrum disorder

ASD refers to a group of specific neurodevelopmental disorders originally termed “early infantile autism” by Kanner in his 1943 case (50). Its core symptoms can be summarized as difficulties in social communication and interaction and restricted and repetitive behaviors, interests, or activities (51). The Global Burden of Disease (GBD) study estimated for the first time that the global prevalence of the disease is approximately 0.76% with no significant regional differences (52). In China, the first national multicenter population-based cross-sectional study of children showed that the prevalence of ASD was approximately 0.70%, with boys being roughly four times more susceptible than girls (53).

**Figure 3:**
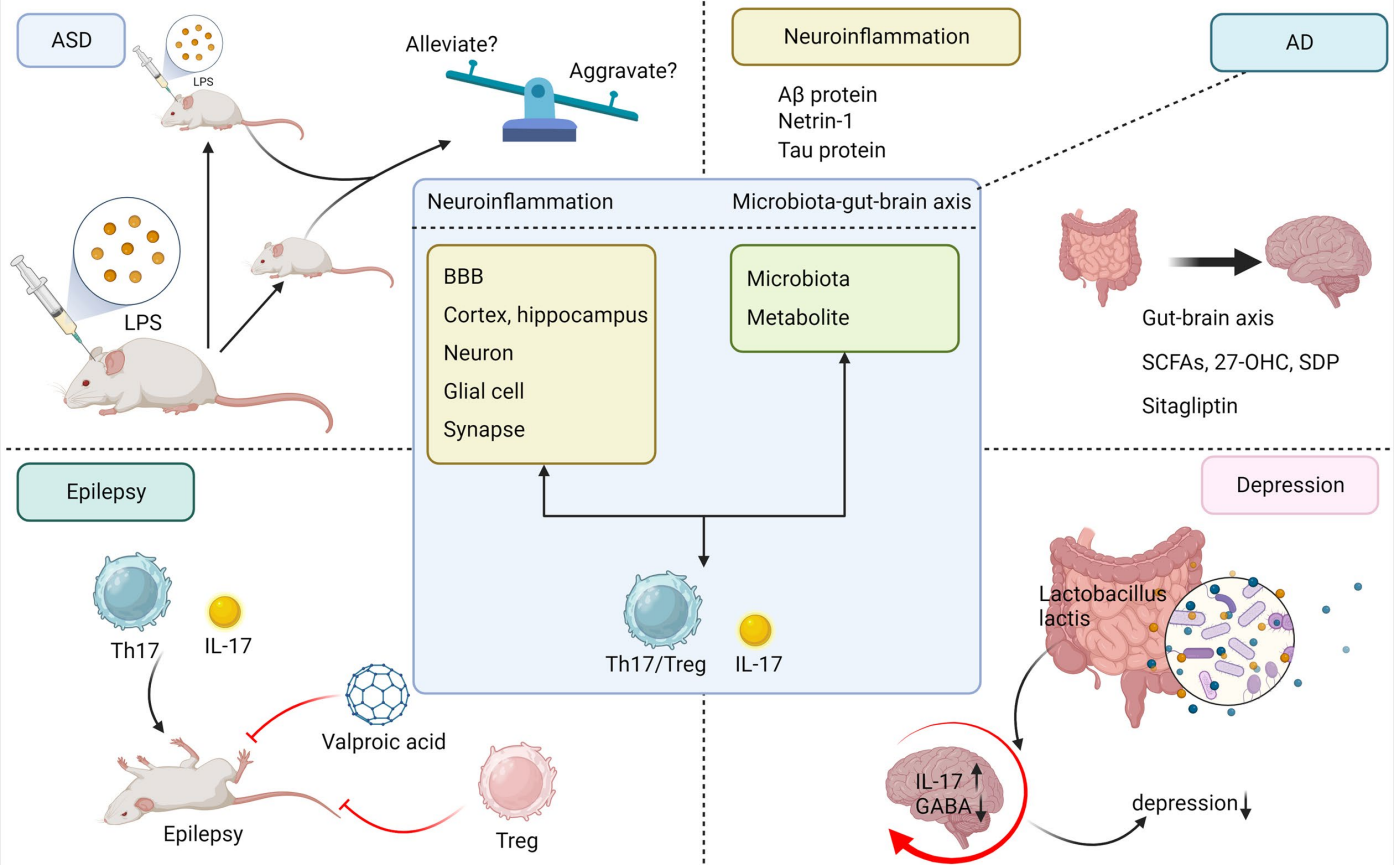
Commonality of IL-17 in neuroinflammation and microbiota–brain–gut axis mechanisms in neuropsychiatric disorders. To summarize the commonalities of IL-17 mediating neuroinflammation, microbiota–gut–brain axis in AD, ASD, epilepsy, and depression and to briefly depict interesting studies between IL-17 and each disease separately. ASD, autism spectrum disorder; AD, Alzheimer’s disease; LPS, lipopolysaccharide; Aβ, amyloid β; 27-OHC, 27-hydroxycholesterol; SDP, spray-dried porcine plasma.

The etiology of ASD is complex and inconclusive, but research suggests that genes and the environment play a leading role (54). The genetic architecture of ASD consists of hundreds or thousands of rare *de novo*, variant genes, or common polygenic risks (55). In genetically susceptible individuals, many environmental risk factors may act synergistically, particularly febrile infections during pregnancy, which may increase the risk of ASD in offspring (56). This is consistent with results observed in animal models of MIA, where offspring of mice with inflammation induced by LPS or poly I:C can exhibit ASD-like behaviors, such as excessive anxiety and reduced socialization (57). Accumulating evidence supports the pronounced nature of IL-17 in ASD. It has been reported that fever temperature significantly enhances the differentiation of Th17 cells and increases their inflammatory capacity (58). In several rodent models of ASD, elevated levels of IL-17 have been observed in serum or tissues of organs such as the uterus and intestine, as well as an increase in IL-17-producing Th17 cells or γδ-T cells (59). Children with ASD have been proven to have elevated serum IL-17 levels compared to healthy children, and the severity of ASD is strongly correlated with the level of IL-17 expression (60). Interestingly, IL-17 can exert very different effects on ASD under different circumstances. Maternal IL-17 acts on the developing brains of offspring mice to induce abnormal cortical phenotypes and promote ASD-like behaviors, and it is required for pregnant mice during MIA-induced behavioral abnormalities in offspring (61). After noting the improvement in ASD-like behavior during fever in a subset of children with ASD, Reed et al. established in a new study that social behavioral deficits in MIA-exposed zygotic mice could be temporarily rescued by the inflammatory response induced by LPS administration and that this rescuing ability was lost by eliminating IL-17RA expression in primary somatosensory cortex dysgranular zone (S1DZ) neurons, suggesting that, during inflammation, IL-17 ameliorates ASD-like symptoms in adult mice (Figure 3) (62). The study may be controversial in that children with ASD manifest stronger and more frequent negative behavioral changes during fever, while a small percentage of children with ASD may be positively affected by fever; however, this is a rare case according to data from the clinical study by Byrne et al. (63).

IL-17 mediates neuroinflammation in ASD. IL-17R is expressed in neuronal, neural stem, astrocytic, microglial, and endothelial cells in the mouse offspring of ASD, and IL-17 signaling can interfere with cortical development through its action on the above cells, leading to the development of cortical dysplasia and behavioral disorders in the offspring linked to ASD (64, 65). Chronic maternal IL-17 was found to cause a persistent ASD-like phenotype in male mouse offspring, accompanied by reduced neuron–neuron synaptic pathway gene expression, cortical volume, and neuroglial density and gene expression, and adult male socialization deficits were negatively correlated with cortical GABAergic-synapse gene expression (66). In the brain tissue of ASD children, astrocyte-derived round membrane vesicles attack lymphocytes, exerting a cytotoxic response that mediates multifocal damage at the perivascular cerebrospinal fluid–brain barrier (67). IL-17 can promote amino acid excitotoxicity via astrocytes (33), and elevated synaptic glutamate levels have been reported in rodent models of ASD and in human patients (68, 69). To assess the effects of IL-17 on cortical development, Sasaki et al. injected IL-17 into the lateral ventricles of the fetal rat brain and observed activated microglia and their accumulation in the cingulate cortex as well as altered localization in the cerebral cortex, resulting in hyperphagocytosis of neural progenitor cells in the ventricular zone of ASD (70). Moreover, IL-17 exacerbated TNF-α-induced oligodendrocytes (OLs) loss and inhibited OL progenitor differentiation (71). After administration of magnesium biotinate (MgB) treatment to the rat model of propionic acid-induced ASD, Purkinje cells in the cerebellum increased in size and density. MgB also reduced brain IL-17, increased serotonin, and improved social behavior, learning, and memory (72). Moreover, IL-17/IL-17R signaling also plays a crucial role in amplifying neuroinflammation via oxidative stress of monocytes. The signaling increases inducible nitric oxide synthase (iNOS)/nitrotyrosine expression in peripheral monocytes of autistic children through the NF-κB pathway, and it may be beneficial for children with ASD to block this signaling pathway (73). It means that IL-17 mediates neuroinflammation not only through the immune cells present within the CNS but also through the monocytes present in the peripheral blood of ASD patients. Subsequently, Nadeem et al. also found that IL-17 and IL-17R signaling in neutrophils coordinate the oxidative inflammatory response with other immune cells, suggesting potential involvement in the neuroinflammatory mechanisms of ASD (74).

Over the years, the role of gut microbiota in alleviating ASD symptoms and the underlying mechanisms has been receiving increasing attention by researchers. When the gut microbiota from ASD patients was transplanted onto germ-free mice, the former was found to induce typical ASD symptoms and lead to aberrant expression and altered splicing of ASD-associated genes in the mouse brain (75). Kim et al. pointed out that pregnant rats treated with SFB or human commensal bacteria, which are capable of inducing Th17 cell differentiation in the gut after gavage colonization, had a significant increase in IL-17 and were more likely to give birth to offspring with ASD-like phenotypes (40). Subsequently, the team further observed that MIA-induced ASD offspring rats were more susceptible to intestinal inflammation after the infection with *Citrobacter rodentium*, and in contrast to the prenatal role of the MIA in neurodevelopmental phenotype, IL-17 mediated an altered naïve CD4+ T chromatin landscape postnatally by modifying the maternal gut microbiota to modulate the offspring’s immune-related phenotype (76). Another study confirmed that Th17 cell differentiation in the lamina propria (LP) of the small intestine is associated with Cytophaga-Flavobacter-Bacteroides phylum (77). In addition to enterobacteria, some metabolites such as the GABA receptor agonist 5-aminovaleric acid (5AV) and taurine significantly ameliorated behavioral abnormalities and modulated neuroexcitability in the mouse brain in ASD mice (75), and GABA signaling affects intestinal IL-17 expression in response to *Citrobacter rodentium* infection in a model of intestinal inflammation (48). In the BTBR mouse model of autism, bile acid deficiency is associated with a reduction in the relative abundance of a very specific group of bacterial taxa, bile-metabolizing *Bifidobacterium, and Blautia* species (78). Bile acid metabolites produced by bacteria, such as 3-oxoLCA, isoLCA, regulate Th17/Treg cell homeostasis and contribute to human gut immune homeostasis (45). SCFAs, conversely, can affect the microbiota–gut–brain axis by restoring microglial defects, with butyrate being particularly strongly associated with ASD. A study of the gut microecology of Chinese children with ASD found a decreased abundance of butyrate-producing bacteria in feces, and this disrupted microbiota was associated with lower levels of fecal butyrate (79). Butyrate, a GM-derived metabolite, downregulates IL-17 expression levels (80) and improves social behavior in ASD mice by modulating the excitatory/inhibitory balance (81).

Prenatal prophylaxis has promising potential in preventing ASD-like behaviors in children born to susceptible mothers, and Choi et al. found that treatment of pregnant dams with antibodies against IL-17A (IL-17Ab) ameliorated MIA-related behavioral abnormalities in offspring; therefore, various related factors in both classical and non-classical IL-17 signaling pathways, such as RORγt and Th17 cells, could serve as effective therapeutic targets (61). Moreover, adoptive cell transfer (ACT) therapies with Treg cells (CD4 + CD25 + Foxp3+) were observed to significantly reverse MIA-induced proinflammatory immune traits and ASD-like abnormal behaviors and rescue anxiety phenotypes and social recognition memory in MIA offspring (82). Gut regulation is equally critical, and modulation of specific IL-17-related flora, lipid metabolism, and neurotransmitters through fecal microbiota transplantation (FMT), oral probiotic capsules, etc. will offer novel insights for clinical translation and large-scale therapies.

### 4.2 IL-17 and Alzheimer’s disease

AD is the most common progressive neurodegenerative disease that mainly affects the elderly, with clinical manifestations of slowly progressive amnesia, aphasia, dysarthria, and dyscognition. It is characterized by intracellular neurofibrillary tangles and extracellular amyloid β-protein (Aβ) plaques as typical pathologic features (83). Aging, genetic factors, head injuries, vascular diseases, infections, and environmental factors are the main risk factors for the disease (84).

Several emerging studies signified a growing emphasis on immune mechanisms in ameliorating or exacerbating the neuropathogenesis of AD (85). In AD patients and AD pre-clinical models, reduced Aβ clearance is associated with BBB damage leading to microglial activation, neuroinflammation, neuronal degeneration, and cognitive deficits (86). In an animal study, BBB disruption by application of Aβ_1-42_ to rat hippocampus resulted in infiltration of Th17 cells into the brain parenchyma, increased hippocampal IL-17 and IL-22 expression, and elevated CSF and serum levels of both cytokines (87). A meta-study based on humans showed that serum IL-17 levels are significantly elevated in AD patients (88), with possible differences between males and females (89). Moreover, *in vivo* administration of IL-17 Abs rescues Aβ-induced neuroinflammation and memory deficits (90). Ye et al. demonstrated that IL-17 signaling promotes neuroinflammation in AD by accelerating the infiltration of CD8+ T lymphocytes and Gr1+CD11b+ myeloid cells (91). The frequency of both IL-17-producing CD4+ and CD8+ cells is significantly increased in AD patients compared to healthy controls (92), and the frequency of Th17 cells is most elevated in the prodromal phase of AD, where their ratio negatively correlates with the CSF Aβ42/Aβ40 ratio (87, 93).

The role of Treg cells is controversial: Their increased numbers may inhibit cognitive deficits by blocking deleterious proinflammatory gliosis or by preventing further demyelination and axonal loss (93, 94); however, another study demonstrated that temporary depletion of the primary stage of Treg cells resulted in the opposite effect, reversing decreased Aβ clearance and cognitive impairment (94). This controversy may have arisen due to the different stages of AD disease in which Treg cell implication was studied (95). Netrin-1 acts as a protective protein with anti-inflammatory and anti-apoptotic effects, and Sun et al. highlighted that a reduction in netrin-1 in AD rats was associated with disruption of the Th17/Treg cell balance, and elevated serum IL-17 and cerebrospinal fluid correlated with disease severity (96). In contrast, using a transgenic 3xTg-AD model, Brigas et al. observed that γδ-T cells accumulate and secrete IL-17 in the meninges of female mice early in the disease, leading to glutamatergic synaptic dysfunction, a process that precedes BBB disruption and Aβ or tau protein lesions. In addition, IL-17 neutralization with a monoclonal antibody is sufficient to prevent cognitive, memory, and synaptic plasticity deficits early in the disease (Figure 3) (97). Research also suggests that 3xTg-AD mice develop neutrophil-dependent brain damage, with neutrophils migrating to the Aβ sediment region and producing IL-17. This directly damages neurons and the BBB and may activate microglia to induce highly deleterious neurotoxic effects, forming a neutrophil-microglia feedback pathway (98).

In recent years, the microbiota–gut–brain axis has received expanding interest in the pathogenesis of AD, and patients with AD can exhibit alterations in both the gut microbiota and blood proinflammatory cytokine profiles. It has been reported that SCFAs from the gut microbiota, particularly propionate, inhibit IL-17 production by murine and human intestinal γδ-T cells (47). Dietary supplementation with SCFAs reduced Aβ deposition and hyperphosphorylation of tau proteins, remodeled microbiota homeostasis, and attenuated cognitive deficits (99). This is due to the fact that SCFAs act on astrocytes to promote glutamate–glutamine shuttling, thereby preventing oxidative neuronal damage (99). Y. Wang et al. noted that cognitive deficits were further exacerbated in AD mice treated with 27-hydroxycholesterol (27-OHC) and that elevated levels of IL-17 in mouse brain and intestinal plasma were associated with mouse ileocecal lesions, changes in intestinal permeability and microbial composition, and decreased levels of SCFAs such as propionate and butyrate (100). A study in aging mice found that dietary supplementation with spray-dried porcine plasma (SDP) as a dietary supplement has both anti-inflammatory properties and the ability to promote the growth of intestinal probiotics. Other capacities include reversing the elevation of IL-17, preventing the increase in expression levels of microglial activation marker genes, and delaying the onset of AD (101). According to recent reports, sitagliptin, a drug that modifies glucose metabolism, exhibited antioxidant and anti-apoptotic properties by altering the levels of glutamate and glutathione in the hippocampus of mice, ameliorating the accumulation of Aβ in the AD brain, and reducing the levels of inflammatory factors such as IL-17 (Figure 3) (102).

Therefore, targeting the neutralization of IL-17, regulation of Th17/Treg cell imbalance, nutrition, microbial therapy, and metabolism-regulating drugs may achieve improvement of AD pathophysiology and alleviation of symptoms, providing a new avenue for clinical treatment of AD.

### 4.3 IL-17 and epilepsy

Epilepsy is a chronic neurological disorder with a tendency to recur unprovoked; epileptic seizures are transient, excessive, abnormal discharges in the brain that temporarily alter behavior and impact more than 65 million people worldwide (103). Brain injury at or after birth, cerebrovascular disease, and CNS infection are the most common risk factors (104). Pathological mechanisms include abnormal ion channel activation, neurotransmitter release, microglial activation, and neuroinflammation (105).

Neuroinflammatory mechanisms have sparked enormous interest in particular, with a 5-fold increased risk of epilepsy in children in most autoimmune diseases (26). As an important cytokine for immune regulation, IL-17 is elevated in brain tissue, cerebrospinal fluid, or serum of epileptic patients with different etiologies (106–109). Its levels may be positively correlated with disease severity, and it may act by penetrating the BBB and increasing neuronal excitability (110, 111). In the studies of epilepsy caused by tuberous sclerosis syndrome (TSC), focal cortical dysplasia (FCD), and medial temporal lobe epilepsy (MTLE), He et al. found that the expression of both IL-17 and IL-17R was upregulated at the mRNA and protein levels by immunofluorescence staining of surgically resected tissues from human patients. Their main sources are the temporal cortex and hippocampal tissue, epileptic cortical nodules, neurons in cortical lesions, pyramidal neurons, granule cells, astrocytes, microglia, and vascular endothelial cells, respectively (112–114). IL-17 promotes glutamate release from astrocytes or inhibits GABA release to induce inhibitory synaptic transmission and increase neuronal excitotoxicity (33, 115). By contrast, Mazdeh et al. detected no significant difference in IL-17 mRNA expression in epilepsy patients compared to healthy subjects or other subgroups (116). In addition, the associated immune cells that influence IL-17 secretion are similarly altered to mediate neuroinflammation. Significantly, γδ-T cells (producing IL-17 and GM-CSF) are concentrated in epileptogenic foci, and their numbers are positively correlated with disease severity (110). The shift of CD4+ T cells to neurotoxic Th17 cells in the peripheral blood of adults with drug-resistant epilepsy (DRE) can lead to a marked increase in serum neurofilament light chain (sNfL), a marker of neuronal damage, particularly in older adults (117). In a model of temporal lobe epilepsy induced by LiCl–pilocarpine, Th17 cells and IL-17 acted mainly in the acute phase, caused necrosis of hippocampal neurons, and positively correlated with neurocognitive dysfunction (118). On the contrary, Treg cells play an immunosuppressive role, mainly during the progressive phase, and Th17 cells and Treg cells limit each other during the recovery phase of epilepsy to achieve functional homeostasis (118). The severity of seizures is exacerbated when Treg cells are overly depleted (110). Ni et al. noticed that Th17/Treg cell imbalance characterizes intractable epilepsy (IE) in children and may contribute to the pathogenesis of IE (119). Balancing Th17 and Treg cells may be an effective treatment for patients with epilepsy (120). However, another study found no significant changes in IL-17 serum concentrations before or after seizures (121).

Studies have shown that the intestinal microbiota of epileptic patients or animals changes and produces various substances that regulate the excitatory-inhibitory balance. These substances enter the CNS alongside damage to the BBB, which appears to be correlated with susceptibility to epileptic activity (122). One study spotted differences in fecal composition between infants with refractory epilepsy and healthy controls (123). The antiepileptic mechanism of probiotics may be related to counteracting neuroinflammation and oxidative stress, and probiotic pretreatment of a mouse model of pentylenetetrazol (PTZ) epilepsy delayed seizures while reversing epilepsy-induced increases in IL-17 and brain oxidative state (124). Epilepsy has a highly overlapping commensal microbiota with type 1 diabetes (T1D), and monocytes from T1DM patients could drive autoimmune epileptogenesis through IL-6 and IL-1β-mediated amplification of Th17 cells (125). SCFAs have been shown to significantly reduce inflammatory parameters such as intestinal IL-17 (126), and De Caro et al. pointed out that intestinal inflammation increases pharmacologically induced seizure activity and that administration of α-lactalbumin (ALAC) and sodium butyrate (NaB) can reduce intestinal inflammation, better restore SCFAs levels, and effectively increase seizure threshold (127).

Therapeutically, the anti-inflammatory properties of the classical antiepileptic drug valproic acid (VPA) can lead to a reduction in IL-17 production (Figure 3) (128). In addition to antiepileptic drugs with antimicrobial activity, antagonists or agonists of IL-17R, exogenous IL-17 stimulation, IL-17 overexpression or knockdown, and immunomodulatory effects of the microbiota offer promising strategies for the treatment of epilepsy.

### 4.4 IL-17 and depression

Depression is a common chronic mental disorder that can seriously compromise the physical and mental health of an individual. Mild depression may manifest as mood and behavior disorders such as sadness, lack of pleasure, worthlessness, and sleep deprivation, and severe depression can lead to recurrent suicidal ideation (129). In recent years, the incidence of depression has been steadily increasing among younger age groups. According to statistics, approximately 34% of adolescents aged 10–19 years worldwide are at risk of developing clinical depression, and this trend is becoming more visible year by year (130).

The molecular mechanisms underlying depression remain unclear with the immune-inflammatory hypothesis gaining prominence as a late focus of depression pathophysiology research. Research has indicated that the proportion of neuroinflammation occurs significantly higher in patients with depression, and the level of inflammatory factors is positively correlated with the degree of depression (131). Patients with first-episode depressive disorder (FDD) have an overactive autoimmune state, and serum IL-17 levels directly affect scoring on the Hamilton rating scale for depression (HAMD) (132). Chen et al. noted that patients with major depressive disorder (MDD) had higher serum concentrations of IL-17 and its specific transcription factor RORγt than controls, accompanied by a significant imbalance in the peripheral Th17/Treg cell ratio (133). IL-17 may be involved in neuroinflammation in depression by disrupting the BBB and targeting neurons and astrocytes or microglia (134). In the LPS-induced depression mouse model, microglia M1 phenotype expression is increased and leads to elevated IL-17, IL-1β, and TNF levels, and endogenous n-3 polyunsaturated fatty acids (PUFAs) may ameliorate depressive-like behaviors by balancing glial cell phenotypes (135). In a study of psoriatic mice, Nadeem et al. found that systemic IL-17 mediates depressive-like behavior by activating the NF-κB/p38MAPK pathway in the mouse brain, leading to the release of associated neuroinflammatory factors in hippocampal and prefrontal cortical regions, and that both anti-IL17 antibodies and NF-κB/p38MAPK inhibitors reduced depressive-like behavior (136). Furthermore, CCR6 knockdown was found to block learned helplessness behavior promoted by T follicular helper 17 (Tfh17) cells aggregated in the hippocampus, acting as an antidepressant (137).

IL-17 can also interact with gut microbes to influence the onset and progression of depression. It has been shown that germ-free mice exhibit reduced anxiety symptoms and that the rebuilding of the gut microbiota early in life partially restores their anxious behavior (134). As early as 1910, a British physician named Phillips proposed that major depression could be treated by taking a gelatin-whey preparation containing lactic acid bacteria (138). Analysis of the microbiome profiles of stress-sensitive mice and patients with major depression revealed a decrease in certain *Lactobacillus* species in the gut microbiota. This was associated with increased differentiation of colonic γδ-T cells mediated by the innate immune receptor Dectin-1 and accumulation of IL-17 in the meninges to promote stress-induced social avoidance behavior, and oral administration of the Dectin-1 ligand porinoside suppressed these changes in the mice (139). Reports stated that *Lactobacillus rhamnosus* JB-1 administration increased GABA expression in the hippocampus of BALB/c mice, with reduced levels of anxiety and corticosterone (140), adding to the evidence that GABA favors the alleviation of depressive symptoms. It is interesting to note that gut mucosa-associated fungi strengthened gut barrier function by increasing IL-17 levels and acted directly on neurons to promote social behavior in mice (Figure 3) (141). Metabolites or neurotransmitters produced by gut microbes act as multifunctional messengers *in vivo* and can modulate stress-related responses and mood disorders. Pistorio et al. identified some differences in lipid metabolism and autonomic dysfunction scores between depressed and healthy individuals (142). It was reported that a high-cholesterol diet (HCD) induced a decrease in the diversity of the intestinal flora, in particular, a decrease in the relative abundance of *Akkermansia muciniphila*. A significant increase in the concentration of serum IL-17 and 5-HT in the hippocampus was also observed, which promoted depressive and anxiety-like behaviors in mice (143). The monoamine neurotransmitter 5-HT can effectively alleviate depressive symptoms and downregulate the expression levels of Th17-related cytokines such as IL-17 and IL-22 in patients with MDD (144). While tryptophan serves as a precursor of 5-HT, the activated tryptophan (Trp) pathway induces the expression of downstream cytokines IL-17 and IL-22, thereby regulating intestinal homeostasis (145).

IL-17 holds the capacity for new treatment options for depression. It can regulate the activation and transduction of the IL-17 signaling pathway at the level of genes, transcription factors, etc., and it is also possible to restore the dysfunctional intestinal ecosystem by personalized supplementation of the aforementioned probiotics related to IL-17 regulation such as *Lactobacillus and Akkermansia muciniphila*, supplemented with prebiotics to boost the proliferation of probiotics (146). Moreover, using FMT and microbiota transfer therapy (MTT), two natural, safe, and promising treatment options to regulate the number, abundance, and diversity of intestinal flora (147) could establish the relationship between IL-17 and key neurotransmitters such as 5-HT and GABA.

### 4.5 Commonality of IL-17 in neuroinflammation, microbiota–brain–gut axis mechanisms in neuropsychiatric disorders

As previously described, IL-17 has been found to mediate the onset, progression, and outcomes through neuroinflammation and the microbiota–gut–brain axis mechanism in ASD, AD, epilepsy, and depression. Basic and clinical studies of IL-17 mediating neuroinflammation in neuropsychiatric disorders are summarized in Tables 1, 2, respectively, and the direct effect of the microbiota–gut–brain axis on IL-17 in such diseases is summarized in Table 3. Moreover, there are intersecting pathways between these two mechanisms. From these findings, we have derived several noteworthy commonalities of IL-17 in the diseases under review in this comprehensive overview.

**Table 1:** Basic studies of IL-17-associated neuroinflammation in neurological diseases and mental disorders.

Diseases	S.N.	Study models	IL-17-related interventions or changes	Etiology	Observations	References
ASD	1	MIA mice	Application of IL-17 during pregnancy	Decreased glial cell density and synaptic genes and increased GABAergic synaptic responses	Chronic gestational IL-17 was sufficient to cause ASD-like phenotypes early and persistently in male offspring.	(66)
	2	MIA mice	Application of IL-17 during pregnancy	Activated microglia and phagocytosed neural progenitor cells	IL-17 activated microglia and altered their localization in the cerebral cortex, leading to a cascade of ASD-related brain pathologies.	(70)
	3	Propionic acid (PPA)-induced ASD rat	IL-17 significantly upregulated in the brain	Destroyed Purkinje Cells in the cerebellum	MgB may ameliorate dysfunctions in social behavior, learning, and memory and reduce inflammatory factors such as brain IL-17 in this rat model.	(72)
AD	4	Aβ_1-42_-induced AD rat	IL-17 increased in the hippocampus, cerebrospinal fluid, and serum	BBB disruption, gliosis, and neuronal apoptosis	Th17 cells release IL-17 and act directly on brain parenchymal neurons via the Fas/FasL apoptotic pathway.	(87)
	5	Aβ_1-42_-induced AD rat	The rat model received IL-17Ab via i.c.v.	Decreased proliferative response and activation of astrocytes and microglia	IL-17Ab reversed neuroinflammation and behavioral symptoms induced by Aβ protein.	(90)
	6	Aβ_1-42_-induced AD rat	IL-17 elevated in both serum and CSF	Substantial amounts of neuronal loss within the hippocampal CA1 region	Decreasing netrin-1 may result in a diminished capacity for immunosuppressive effects on Th17/Treg cells.	(96)
	7	3xTg-AD mice	IL-17 increased in meninges, brain, and cLNs of female	Glutamatergic synaptic dysfunction	Elevated levels of IL-17 at early stages of disease contribute to synaptic dysfunction and short-term memory deficits.	(97)
	8	3xTg-AD mice	IL-17 increased in the choroid plexuses, cortex, and hippocampus	Directly toxic to neurons and the BBB and may recruit more neutrophils	Neutrophils release neutrophil extracellular traps and IL-17 in areas of Aβ deposits, which contribute to pathogenesis and cognitive impairment of AD.	(98)
	9	Hybrid mouse with IL-17RA knockout and APP/PS1	IL-17RA knockout	Decreased infiltration of CD8+ T cells and myeloid cells to mouse brain	The increase of IL-17 producers is associated with short-term memory deficits; IL-17Ab prevents cognitive impairments and synaptic dysfunction.	(91)
Epilepsy	10	Kainic acid model mice of status epilepticus	IL-17-producing γδ-T cells were concentrated in the brain	Induced neuronal hyperexcitability and compromised cell viability in an extended time period in brain slice cultures	IL-17RA KO mice displayed significantly delayed seizure onset, the neurons recorded from IL-17 slices exhibited significantly enhanced excitability, and prolonged exposure to IL-17 causes neuronal apoptosis.	(110)
	11	LiCl–pilocarpine-induced temporal lobe epilepsy (TLE) rat models	IL-17 and Th17 cells were significantly increased	Induced to hippocampal neuron necrosis, damage, and decrease	Th17 cells and IL-17 play a role in the acute phase of epilepsy and are positively correlated with hippocampal lesions and neurocognitive dysfunction.	(118)
Depression	12	LPS-induced depressed mice	IL-17 increased in the hippocampus	Increases microglia M1 type and inhibits M2 type-mediated neuroinflammation	Endogenous n-3 PUFAs may improve depression by balancing microglial M1 and M2-phenotypes to reduce proinflammatory cytokines such as IL-17.	(135)
	13	IMQ-induced psoriasis-like skin inflammation in mice	Treating mice with recombinant mouse IL-17 i.p	Hippocampal NF-κB/p38MAPK signaling pathway mediates neuroinflammation	IL17A/IL-17RA signaling activated by psoriatic inflammation in the brain mediates downstream inflammatory effects required for depression.	(136)

**Table 2:** Clinical studies of IL-17-associated neuroinflammation in neurological diseases and mental disorders.

Diseases	S.N.	Subjects	IL-17-related interventions or changes	Etiology	Observations	References
ASD	1	ASD children	Increased IL-17A/IL-17RA signaling in monocytes of ASD children	Increased oxidative stress, increased NF-κB pathway, and iNOS/nitrotyrosine expression	Enhanced IL-17A/IL-17RA signaling is associated with enhanced oxidative inflammation in monocytes, which may exacerbate neuroinflammation, and blocking the signaling may be beneficial in ASD children.	(73)
	2	ASD children	Increased IL-17A and IL-17R expressions in neutrophils of ASD children	Increased oxidative, inflammatory mediators and NF-kB, NOX2/ROS, and IL-6 signaling	In ASD children, IL-17A and IL-17RA are upregulated, which is associated with increased oxidative, inflammatory responses, and aforementioned signaling in neutrophils.	(74)
AD	3	AD patients	IL-17 and Th17 cells were significantly elevated	BBB disruption, gliosis, and neuronal apoptosis	The increase of Th17 cells and the association of Treg cells with neurodegeneration marker Tau may indicate that the adaptive immune system relates to the neuroinflammation of AD.	(93)
Epilepsy	4	Pediatric epilepsy patients	IL-17-producing γδ-T cells were concentrated in the epileptic lesion center of epilepsy children	Neuronal excitability, dysfunction and death	The number of IL-17-producing γδ-T cells in the brain of epileptic patients positively correlated with seizure frequency, and Treg cells negatively correlated with seizure frequency.	(110)
	5	Patients undergoing surgery for intractable epilepsy (TSC, FCD, MTLE)	IL-17 and IL-17R were upregulated at both mRNA and protein levels in brain tissues obtained from surgery	Involvement of neurons, astrocytes, microglia, and endothelial cells of blood vessels	IL-17 is overexpressed in intractable epilepsy, the aforementioned cells are the main source of IL-17, and IL-17 may promote BBB disruption and inflammation.	(112–114)
	6	Patients classified by neurologist as DRE	IL-17 was elevated in the serum	BBB destabilization and neuronal hyper-excitability, and reduced neuroprotection	Th17 cells and IL-17 are clearly increased in DRE, and pathological levels of sNfL are increased, supporting an underlying neurodegenerative component.	(117)
Depression	**-**	-	-	-	-	-

**Table 3:** Direct impact of the microbiota–gut–brain axis on IL-17 in neurological diseases and mental disorders.

S.N.	Diseases	Etiology	Treatment	Effects on the level of IL-17	Risk of disease or changes in symptoms	References
1	ASD	Microbiota	SFB	↑	Increased risk in ASD	(40)
2	ASD	Microbiota	*Citrobacter rodentium*	↑	Increased risk of developing inflammatory diseases	(76)
3	AD	Microbiota	SDP	↓	Alleviated symptoms of AD	(101)
4	AD	Microbiota, Metabolic	27-OHC	↑	Aggravated symptoms of AD	(100)
5	AD	Metabolic, Neurotransmitter	Sitagliptin	↓	Alleviated symptoms of AD	(102)
6	Epilepsy	Microbiota	Probiotic mixture supplementation	↓	Alleviated symptoms of epilepsy	(124)
7	Depression	Microbiota	Pachyman	↓	Alleviated symptoms of depression	(139)
8	Depression	Microbiota, Metabolic, Neurotransmitter	High-cholesterol diet (HCD)	↓	Aggravated symptoms of depression	(143)
9	Depression	Neurotransmitter	5-HT	↓	Alleviated symptoms of depression	(144)

First and foremost, commonalities observed in IL-17-related neuroinflammation are particularly striking. As early as 2007, research conducted on the animal model of MS, EAE mice, revealed that IL-17 induces BBB damage (148). These four diseases described in this review also validate this point. Once the integrity of the BBB is compromised, a greater influx of Th17 cells, neutrophils, or δβ-T cells occurs within the brain parenchyma, consequently leading to an upsurge in IL-17 production and the initiation of irreversible neuronal dysfunction (98, 110, 122, 134). After IL-17 infiltration into the CNS, the cerebral cortex is a primary site that may be subject to its regulatory influence. Notably, in the context of autism, IL-17’s role in impairing cortical development is more evident (61, 64, 65). It may also affect the positioning of microglial cells and synaptic function within this region (66, 70). And in the cases of epilepsy and depression, IL-17 predominantly exerts proinflammatory characteristics within the cortex, mediating the release of various inflammatory factors (112–114, 136). The hippocampus, being part of the limbic system and closely associated with memory and cognitive functions (149), demonstrates upregulation of IL-17 in AD, epilepsy, and depression (87, 112–114, 137). Furthermore, it may be crucially linked to the levels of neurotransmitters such as glutamate, GABA, and 5-HT, indirectly modulating depressive, anxiety-like emotions, and improving cognitive function (102, 140, 143). As the fundamental structural and functional unit of the nervous system, neurons are regulated by IL-17 in all the diseases discussed in this study. Dysregulation of IL-17 can induce neuronal damage and even apoptosis at the lesion site (64, 65, 98, 112–114, 134, 141). Notably, IL-17’s impact on neurons in epilepsy is distinctive compared to other diseases as it can provoke excitotoxicity in neurons (110, 111). Furthermore, it can modulate the expression of excitatory and inhibitory neurotransmitters through astrocytes (33, 115), potentially supplementing the mechanisms underlying neurotransmitter imbalance and seizure initiation (150).

IL-17 also mediates the microbiota–gut–brain axis, which collectively participates in the pathophysiological mechanisms of the diseases discussed in this review. We propose that astrocytes and microglial cells can be regarded as central hubs in IL-17-induced ‘neuroinflammation and microbiota–gut–brain axis’ crosstalk. They play a crucial regulatory role in processes such as neuronal growth, differentiation, and the formation and pruning of synapses (151). IL-17 not only directly influences the expression and differentiation of glial cells (64–66, 112–114, 135), but it is worth noting that it also has indirect regulatory connections with the microbiota or metabolites such as SCFAs and n-3 PUFAS, which can further induce neurotoxicity in glial cells (33, 98, 99, 101, 135). Synapses are crucial sites for information transmission in the nervous system, and IL-17 can inhibit the transmission at GABAergic synapses (115). In ASD, IL-17 reduces GABA release by suppressing the gene expression at GABAergic synapses, contributing to disease alleviation (66). In depression, the administration of JB-1 can regulate gut microbiota composition, promote GABA expression, and alleviate anxiety levels (140). In AD, elevated IL-17 levels can lead to dysfunction in glutamatergic synapses (97), and it can also indirectly participate in the regulation of glutamate metabolism through SCFAs (99). Furthermore, increased glutamatergic synaptic activity has been detected in both human and animal models of ASD (68, 69). All of these findings provide insights into how IL-17 regulates social behavior, cognitive levels, anxiety manifestations, and more through the crosstalk between neuroinflammation and the microbiota–gut–brain axis.

Finally, whether the above diseases are regulated by neuroinflammatory or microbiota–brain–gut axis mechanisms, the imbalance of the Th17/Treg cell ratio is more significant in terms of changes in cellular phenotypes (96, 119, 133). The degree of imbalance may be positively correlated with disease severity (96), and improving the imbalanced Th17/Treg cell ratio may be one of the targets for therapy (119). In Figure 3, we depict the commonalities through which IL-17 mediates the four diseases via the aforementioned mechanisms, incorporating interesting related research findings for each condition.

## 5 Conclusion

In summary, with IL-17 as a widely discussed inflammatory target, an increasing body of research supports its crucial role in mediating immune regulation through neuroinflammation and the microbiota–gut–brain axis in common neurological diseases and mental disorders. Although clinical and animal studies have started to uncover how IL-17 affects ASD, AD, epilepsy, and depression, it is debatable whether the outcomes of animal models can be directly applicable to humans. This is due to the complexity of the human neurological system at work and the fact that the gut microbiota and its metabolism are subject to a myriad of factors such as genetics, ethnicity, age, and lifestyle; therefore, the involvement of IL-17 in the pathogenesis of disease and the crosstalk and interaction of various pathways are remains to be explored.

Currently, numerous IL-17-targeted inhibitors have been approved for the treatment of autoimmune diseases such as psoriasis, rheumatoid arthritis, and ankylosing spondylitis or for those that have advanced to phase III clinical trials. Moving forward, a combination of techniques and research directions can be implemented to inform future studies. This may include multifaceted regulation of Th17/Treg cells, IL-17 signaling pathway and related factors, and IL-17-related glial cells, emphasizing the role of microbiota–gut–brain axis in the regulation of immunity. Additionally, adapting therapies such as dietary modification, neurotransmitter supplementation, specific probiotics, and FMT derived from healthy volunteers may provide new perspectives on immunity and the treatment of neurological diseases and mental disorders.

## Author contributions

YL: Writing – original draft, Conceptualization, Supervision, Visualization. PZ: Writing – original draft. FX: Writing – original draft. YZ: Writing – original draft. HZ: Writing – review & editing.

## References

[1] Schinocca C Rizzo C Fasano S Grasso G La Barbera L Ciccia F. Role of the IL-23/IL-17 pathway in rheumatic diseases: An overview. Front Immunol. (2021) 12:637829. doi: 10.3389/fimmu.2021.637829, PMID: 33692806 PMC7937623

[2] Patel S Dale RC Rose D Heath B Nordahl CW Rogers S. Maternal immune conditions are increased in males with autism spectrum disorders and are associated with behavioural and emotional but not cognitive co-morbidity. Transl Psychiatry. (2020) 10:286. doi: 10.1038/s41398-020-00976-2 32796821 PMC7429839

[3] GBD 2019 Mental Disorders Collaborators. Global, regional, and national burden of 12 mental disorders in 204 countries and territories, 1990-2019: a systematic analysis for the global burden of disease study 2019. Lancet Psychiatry. (2022) 9:137–50. doi: 10.1016/S2215-0366(21)00395-3, PMID: 35026139 PMC8776563

[4] Leng F Edison P. Neuroinflammation and microglial activation in Alzheimer disease: where do we go from here? Nat Rev Neurol. (2021) 17:157–72. doi: 10.1038/s41582-020-00435-y, PMID: 33318676

[5] Han VX Patel S Jones HF Dale RC. Maternal immune activation and neuroinflammation in human neurodevelopmental disorders. Nat Rev Neurol. (2021) 17:564–79. doi: 10.1038/s41582-021-00530-8, PMID: 34341569

[6] Pellegrini C Fornai M D’Antongiovanni V Antonioli L Bernardini N Derkinderen P. The intestinal barrier in disorders of the central nervous system. Lancet Gastroenterol Hepatol. (2023) 8:66–80. doi: 10.1016/S2468-1253(22)00241-2, PMID: 36334596

[7] Rouvier E Luciani MF Mattéi MG Denizot F Golstein P. CTLA-8, cloned from an activated T cell, bearing AU-rich messenger RNA instability sequences, and homologous to a herpesvirus saimiri gene. J Immunol. (1993) 150:5445–56. doi: 10.4049/jimmunol.150.12.5445, PMID: 8390535

[8] Shibabaw T. Inflammatory cytokine: IL-17A signaling pathway in patients present with COVID-19 and current treatment strategy. J Inflamm Res. (2020) 13:673–80. doi: 10.2147/JIR.S278335 33116747 PMC7547786

[9] McGeachy MJ Cua DJ Gaffen SL. The IL-17 family of cytokines in health and disease. Immunity. (2019) 50:892–906. doi: 10.1016/j.immuni.2019.03.021, PMID: 30995505 PMC6474359

[10] Gaffen SL. Structure and signalling in the IL-17 receptor family. Nat Rev Immunol. (2009) 9:556–67. doi: 10.1038/nri2586, PMID: 19575028 PMC2821718

[11] Amatya N Garg AV Gaffen SL. IL-17 signaling: the Yin and the Yang. Trends Immunol. (2017) 38:310–22. doi: 10.1016/j.it.2017.01.006, PMID: 28254169 PMC5411326

[12] Gaffen SL. Life before seventeen: cloning of the IL-17 receptor. J Immunol. (2011) 187:4389–91. doi: 10.4049/jimmunol.1102576, PMID: 22013204 PMC4824884

[13] Douzandeh-Mobarrez B Kariminik A. Gut microbiota and IL-17A: physiological and pathological responses. Probiotics Antimicrob Proteins. (2019) 11:1–10. doi: 10.1007/s12602-017-9329-z, PMID: 28921400

[14] Mills KHG. IL-17 and IL-17-producing cells in protection versus pathology. Nat Rev Immunol. (2023) 23:38–54. doi: 10.1038/s41577-022-00746-9, PMID: 35790881 PMC9255545

[15] Park H Li Z Yang XO Chang SH Nurieva R Wang Y-H. A distinct lineage of CD4 T cells regulates tissue inflammation by producing interleukin 17. Nat Immunol. (2005) 6:1133–41. doi: 10.1038/ni1261, PMID: 16200068 PMC1618871

[16] Moser T Akgün K Proschmann U Sellner J Ziemssen T. The role of TH17 cells in multiple sclerosis: therapeutic implications. Autoimmun Rev. (2020) 19:102647. doi: 10.1016/j.autrev.2020.102647, PMID: 32801039

[17] Gaffen SL Jain R Garg AV Cua DJ. IL-23-IL-17 immune axis: discovery, mechanistic understanding, and clinical testing. Nat Rev Immunol. (2014) 14:585–600. doi: 10.1038/nri3707, PMID: 25145755 PMC4281037

[18] Capone A Volpe E. Transcriptional regulators of T helper 17 cell differentiation in health and autoimmune diseases. Front Immunol. (2020) 11:348. doi: 10.3389/fimmu.2020.00348 32226427 PMC7080699

[19] Zhang W Liu X Zhu Y Liu X Gu Y Dai X. Transcriptional and posttranslational regulation of Th17/Treg balance in health and disease. Eur J Immunol. (2021) 51:2137–50. doi: 10.1002/eji.202048794, PMID: 34322865

[20] Moaaz M Youssry S Elfatatry A El Rahman MA. Th17/Treg cells imbalance and their related cytokines (IL-17, IL-10 and TGF-β) in children with autism spectrum disorder. J Neuroimmunol. (2019) 337:577071. doi: 10.1016/j.jneuroim.2019.577071, PMID: 31671361

[21] Kluger MA Melderis S Nosko A Goerke B Luig M Meyer MC. Treg17 cells are programmed by Stat3 to suppress Th17 responses in systemic lupus. Kidney Int. (2016) 89:158–66. doi: 10.1038/ki.2015.296, PMID: 26466322

[22] Miossec P Kolls JK. Targeting IL-17 and TH17 cells in chronic inflammation. Nat Rev Drug Discov. (2012) 11:763–76. doi: 10.1038/nrd3794, PMID: 23023676

[23] Miyashita Y Kouwaki T Tsukamoto H Okamoto M Nakamura K Oshiumi H. TICAM-1/TRIF associates with Act1 and suppresses IL-17 receptor–mediated inflammatory responses. Life Sci Alliance. (2021) 5:e202101181. doi: 10.26508/lsa.202101181 34819358 PMC8616538

[24] Sun E Motolani A Campos L Lu T. The pivotal role of NF-kB in the pathogenesis and therapeutics of Alzheimer’s disease. Int J Mol Sci. (2022) 23:8972. doi: 10.3390/ijms23168972, PMID: 36012242 PMC9408758

[25] Gu C Wu L Li X. IL-17 family: cytokines, receptors and signaling. Cytokine. (2013) 64:477–85. doi: 10.1016/j.cyto.2013.07.022, PMID: 24011563 PMC3867811

[26] Pracucci E Pillai V Lamers D Parra R Landi S. Neuroinflammation: a signature or a cause of epilepsy? Int J Mol Sci. (2021) 22:6981. doi: 10.3390/ijms22136981, PMID: 34209535 PMC8267969

[27] Liu Z Qiu A-W Huang Y Yang Y Chen J-N Gu T-T. IL-17A exacerbates neuroinflammation and neurodegeneration by activating microglia in rodent models of Parkinson’s disease. Brain Behav Immun. (2019) 81:630–45. doi: 10.1016/j.bbi.2019.07.026, PMID: 31351185

[28] Sun J Zhang S Zhang X Zhang X Dong H Qian Y. IL-17A is implicated in lipopolysaccharide-induced neuroinflammation and cognitive impairment in aged rats via microglial activation. J Neuroinflammation. (2015) 12:165. doi: 10.1186/s12974-015-0394-5, PMID: 26373740 PMC4572693

[29] Lv M Liu Y Zhang J Sun L Liu Z Zhang S. Roles of inflammation response in microglia cell through toll-like receptors 2/interleukin-23/interleukin-17 pathway in cerebral ischemia/reperfusion injury. Neuroscience. (2011) 176:162–72. doi: 10.1016/j.neuroscience.2010.11.066, PMID: 21182899

[30] Yan Y Ding X Li K Ciric B Wu S Xu H. CNS-specific therapy for ongoing EAE by silencing IL-17 pathway in astrocytes. Mol Ther. (2012) 20:1338–48. doi: 10.1038/mt.2012.12, PMID: 22434134 PMC3392982

[31] Linnerbauer M Wheeler MA Quintana FJ. Astrocyte crosstalk in CNS inflammation. Neuron. (2020) 108:608–22. doi: 10.1016/j.neuron.2020.08.012, PMID: 32898475 PMC7704785

[32] Lin Y Zhang J-C Yao C-Y Wu Y Abdelgawad AF Yao S-L. Critical role of astrocytic interleukin-17 a in post-stroke survival and neuronal differentiation of neural precursor cells in adult mice. Cell Death Dis. (2016) 7:e2273. doi: 10.1038/cddis.2015.284, PMID: 27336717 PMC5143370

[33] Kostic M Zivkovic N Cvetanovic A Stojanovic I Colic M. IL-17 signalling in astrocytes promotes glutamate excitotoxicity: indications for the link between inflammatory and neurodegenerative events in multiple sclerosis. Mult Scler Relat Disord. (2017) 11:12–7. doi: 10.1016/j.msard.2016.11.006, PMID: 28104249

[34] Wang D Zhao Y Wang G Sun B Kong Q Zhao K. IL-17 potentiates neuronal injury induced by oxygen-glucose deprivation and affects neuronal IL-17 receptor expression. J Neuroimmunol. (2009) 212:17–25. doi: 10.1016/j.jneuroim.2009.04.007, PMID: 19457561

[35] Cui L Xue R Zhang X Chen S Wan Y Wu W. Sleep deprivation inhibits proliferation of adult hippocampal neural progenitor cells by a mechanism involving IL-17 and p38 MAPK. Brain Res. (2019) 1714:81–7. doi: 10.1016/j.brainres.2019.01.024, PMID: 30677408

[36] Tfilin M Turgeman G. Interleukine-17 administration modulates adult hippocampal neurogenesis and improves spatial learning in mice. J Mol Neurosci. (2019) 69:254–63. doi: 10.1007/s12031-019-01354-4, PMID: 31254254

[37] Sherwin E Bordenstein SR Quinn JL Dinan TG Cryan JF. Microbiota and the social brain. Science. (2019) 366:eaar2016. doi: 10.1126/science.aar2016 31672864

[38] Donaldson GP Lee SM Mazmanian SK. Gut biogeography of the bacterial microbiota. Nat Rev Microbiol. (2016) 14:20–32. doi: 10.1038/nrmicro3552, PMID: 26499895 PMC4837114

[39] Kawano Y Edwards M Huang Y Bilate AM Araujo LP Tanoue T. Microbiota imbalance induced by dietary sugar disrupts immune-mediated protection from metabolic syndrome. Cells. (2022) 185:3501–19. doi: 10.1016/j.cell.2022.08.005, PMID: 36041436 PMC9556172

[40] Kim S Kim H Yim YS Ha S Atarashi K Tan TG. Maternal gut bacteria promote neurodevelopmental abnormalities in mouse offspring. Nature. (2017) 549:528–32. doi: 10.1038/nature23910, PMID: 28902840 PMC5870873

[41] Regen T Isaac S Amorim A Núñez NG Hauptmann J Shanmugavadivu A. IL-17 controls central nervous system autoimmunity through the intestinal microbiome. Sci Immunol. (2021) 6:eaaz6563. doi: 10.1126/sciimmunol.aaz6563 33547052

[42] Majumder S McGeachy MJ. IL-17 in the pathogenesis of disease: good intentions gone awry. Annu Rev Immunol. (2021) 39:537–56. doi: 10.1146/annurev-immunol-101819-092536, PMID: 33577346 PMC8603601

[43] Benakis C Brea D Caballero S Faraco G Moore J Murphy M. Commensal microbiota affects ischemic stroke outcome by regulating intestinal γδ T cells. Nat Med. (2016) 22:516–23. doi: 10.1038/nm.4068, PMID: 27019327 PMC4860105

[44] Bechara R McGeachy MJ Gaffen SL. The metabolism-modulating activity of IL-17 signaling in health and disease. J Exp Med. (2021) 218:e20202191. doi: 10.1084/jem.20202191, PMID: 33822846 PMC8025242

[45] Hang S Paik D Yao L Kim E Trinath J Lu J. Bile acid metabolites control TH17 and Treg cell differentiation. Nature. (2019) 576:143–8. doi: 10.1038/s41586-019-1785-z, PMID: 31776512 PMC6949019

[46] Hammer A Schliep A Jörg S Haghikia A Gold R Kleinewietfeld M. Impact of combined sodium chloride and saturated long-chain fatty acid challenge on the differentiation of T helper cells in neuroinflammation. J Neuroinflammation. (2017) 14:184. doi: 10.1186/s12974-017-0954-y 28899400 PMC5596846

[47] Dupraz L Magniez A Rolhion N Richard ML Da Costa G Touch S. Gut microbiota-derived short-chain fatty acids regulate IL-17 production by mouse and human intestinal γδ T cells. Cell Rep. (2021) 36:109332. doi: 10.1016/j.celrep.2021.109332, PMID: 34233192

[48] Ren W Yin J Xiao H Chen S Liu G Tan B. Intestinal microbiota-derived GABA mediates Interleukin-17 expression during Enterotoxigenic *Escherichia coli* infection. Front Immunol. (2016) 7:685. doi: 10.3389/fimmu.2016.00685, PMID: 28138329 PMC5237640

[49] Brambilla P Perez J Barale F Schettini G Soares JC. GABAergic dysfunction in mood disorders. Mol Psychiatry. (2003) 8:721–37. doi: 10.1038/sj.mp.4001362 12888801

[50] Nelson RH. Autism advocacy before and after DSM-5. Am J Bioeth. (2020) 20:48–50. doi: 10.1080/15265161.2020.1730506, PMID: 32223630

[51] Lai M-C Lombardo MV Baron-Cohen S. Autism. Lancet. (2014) 383:896–910. doi: 10.1016/S0140-6736(13)61539-1, PMID: 24074734

[52] Baxter AJ Brugha TS Erskine HE Scheurer RW Vos T Scott JG. The epidemiology and global burden of autism spectrum disorders. Psychol Med. (2015) 45:601–13. doi: 10.1017/S003329171400172X, PMID: 25108395

[53] Zhou H Xu X Yan W Zou X Wu L Luo X. Prevalence of autism Spectrum disorder in China: a Nationwide multi-center population-based study among children aged 6 to 12 years. Neurosci Bull. (2020) 36:961–71. doi: 10.1007/s12264-020-00530-6, PMID: 32607739 PMC7475160

[54] Hirota T King BH. Autism Spectrum disorder: a review. JAMA. (2023) 329:157–68. doi: 10.1001/jama.2022.23661, PMID: 36625807

[55] Iakoucheva LM Muotri AR Sebat J. Getting to the cores of autism. Cells. (2019) 178:1287–98. doi: 10.1016/j.cell.2019.07.037, PMID: 31491383 PMC7039308

[56] Brucato M Ladd-Acosta C Li M Caruso D Hong X Kaczaniuk J. Prenatal exposure to fever is associated with autism Spectrum disorder in the Boston birth cohort. Autism Res. (2017) 10:1878–90. doi: 10.1002/aur.1841, PMID: 28799289 PMC5685874

[57] Fernández de Cossío L Guzmán A van der Veldt S Luheshi GN. Prenatal infection leads to ASD-like behavior and altered synaptic pruning in the mouse offspring. Brain Behav Immun. (2017) 63:88–98. doi: 10.1016/j.bbi.2016.09.028, PMID: 27697456

[58] Wang X Ni L Wan S Zhao X Ding X Dejean A. Febrile temperature critically controls the differentiation and pathogenicity of T helper 17 cells. Immunity. (2020) 52:328–341.e5. doi: 10.1016/j.immuni.2020.01.006, PMID: 32049050

[59] Thawley AJ Veneziani LP Rabelo-da-Ponte FD Riederer I Mendes-da-Cruz DA Bambini-Junior V. Aberrant IL-17 levels in rodent models of autism Spectrum disorder: a systematic review. Front Immunol. (2022) 13:874064. doi: 10.3389/fimmu.2022.874064, PMID: 35757754 PMC9226456

[60] Al-Ayadhi LY Mostafa GA. Elevated serum levels of interleukin-17A in children with autism. J Neuroinflammation. (2012) 9:158. doi: 10.1186/1742-2094-9-158, PMID: 22748016 PMC3410815

[61] Choi GB Yim YS Wong H Kim S Kim H Kim SV. The maternal interleukin-17a pathway in mice promotes autismlike phenotypes in offspring. Science. (2016) 351:933–9. doi: 10.1126/science.aad0314, PMID: 26822608 PMC4782964

[62] Reed MD Yim YS Wimmer RD Kim H Ryu C Welch GM. IL-17a promotes sociability in mouse models for neurodevelopmental disorders. Nature. (2020) 577:249–53. doi: 10.1038/s41586-019-1843-6, PMID: 31853066 PMC8112727

[63] Byrne K Zheng S Bishop S Boucher J Ghods S Kim SH. Behavioral responses to fevers and other medical events in children with and without ASD. Autism Res. (2022) 15:2056–63. doi: 10.1002/aur.2810, PMID: 36164255

[64] Ozaki K Kato D Ikegami A Hashimoto A Sugio S Guo Z. Maternal immune activation induces sustained changes in fetal microglia motility. Sci Rep. (2020) 10:21378. doi: 10.1038/s41598-020-78294-2 33288794 PMC7721716

[65] Wong H Hoeffer C. Maternal IL-17A in autism. Exp Neurol. (2018) 299:228–40. doi: 10.1016/j.expneurol.2017.04.010, PMID: 28455196 PMC5656543

[66] Gumusoglu SB Hing BWQ Chilukuri ASS Dewitt JJ Scroggins SM Stevens HE. Chronic maternal interleukin-17 and autism-related cortical gene expression, neurobiology, and behavior. Neuropsychopharmacology. (2020) 45:1008–17. doi: 10.1038/s41386-020-0640-0, PMID: 32074626 PMC7162858

[67] DiStasio MM Nagakura I Nadler MJ Anderson MP. T lymphocytes and cytotoxic astrocyte blebs correlate across autism brains. Ann Neurol. (2019) 86:885–98. doi: 10.1002/ana.25610, PMID: 31591744 PMC7210715

[68] Smith SEP Zhou Y-D Zhang G Jin Z Stoppel DC Anderson MP. Increased gene dosage of Ube3a results in autism traits and decreased glutamate synaptic transmission in mice. Sci Transl Med. (2011) 3:103ra97. doi: 10.1126/scitranslmed.3002627 PMC3356696 21974935

[69] Purcell AE Jeon OH Zimmerman AW Blue ME Pevsner J. Postmortem brain abnormalities of the glutamate neurotransmitter system in autism. Neurology. (2001) 57:1618–28. doi: 10.1212/WNL.57.9.1618, PMID: 11706102

[70] Sasaki T Tome S Takei Y. Intraventricular IL-17A administration activates microglia and alters their localization in the mouse embryo cerebral cortex. Mol Brain. (2020) 13:93. doi: 10.1186/s13041-020-00635-z 32546246 PMC7298827

[71] Paintlia MK Paintlia AS Singh AK Singh I. Synergistic activity of interleukin-17 and tumor necrosis factor-α enhances oxidative stress-mediated oligodendrocyte apoptosis. J Neurochem. (2011) 116:508–21. doi: 10.1111/j.1471-4159.2010.07136.x, PMID: 21143599 PMC3033460

[72] Sahin K Orhan C Karatoprak S Tuzcu M Deeh PBD Ozercan IH. Therapeutic effects of a novel form of biotin on propionic acid-induced autistic features in rats. Nutrients. (2022) 14:1280. doi: 10.3390/nu14061280, PMID: 35334937 PMC8955994

[73] Nadeem A Ahmad SF Attia SM Bakheet SA Al-Harbi NO AL-Ayadhi LY. Activation of IL-17 receptor leads to increased oxidative inflammation in peripheral monocytes of autistic children. Brain Behav Immun. (2018) 67:335–44. doi: 10.1016/j.bbi.2017.09.010, PMID: 28935156

[74] Nadeem A Ahmad SF Attia SM Al-Ayadhi LY Bakheet SA Al-Harbi NO. Oxidative and inflammatory mediators are upregulated in neutrophils of autistic children: role of IL-17A receptor signaling. Prog Neuro-Psychopharmacol Biol Psychiatry. (2019) 90:204–11. doi: 10.1016/j.pnpbp.2018.12.002 30529000

[75] Sharon G Cruz NJ Kang D-W Gandal MJ Wang B Kim Y-M. Human gut microbiota from autism Spectrum disorder promote behavioral symptoms in mice. Cells. (2019) 177:1600–1618.e17. doi: 10.1016/j.cell.2019.05.004, PMID: 31150625 PMC6993574

[76] Kim E Paik D Ramirez RN Biggs DG Park Y Kwon H-K. Maternal gut bacteria drive intestinal inflammation in offspring with neurodevelopmental disorders by altering the chromatin landscape of CD4+ T cells. Immunity. (2022) 55:145–158.e7. doi: 10.1016/j.immuni.2021.11.005, PMID: 34879222 PMC8755621

[77] Ivanov II de Llanos FR Manel N Yoshinaga K Rifkin DB Sartor RB. Specific microbiota direct the differentiation of Th17 cells in the mucosa of the small intestine. Cell Host Microbe. (2008) 4:337–49. doi: 10.1016/j.chom.2008.09.009, PMID: 18854238 PMC2597589

[78] Golubeva AV Joyce SA Moloney G Burokas A Sherwin E Arboleya S. Microbiota-related changes in Bile Acid & Tryptophan Metabolism are associated with gastrointestinal dysfunction in a mouse model of autism. EBioMedicine. (2017) 24:166–78. doi: 10.1016/j.ebiom.2017.09.020, PMID: 28965876 PMC5652137

[79] Liu S Li E Sun Z Fu D Duan G Jiang M. Altered gut microbiota and short chain fatty acids in Chinese children with autism spectrum disorder. Sci Rep. (2019) 9:287. doi: 10.1038/s41598-018-36430-z 30670726 PMC6342986

[80] Fernando MR Saxena A Reyes J-L McKay DM. Butyrate enhances antibacterial effects while suppressing other features of alternative activation in IL-4-induced macrophages. Am J Physiol Gastrointest Liver Physiol. (2016) 310:G822–31. doi: 10.1152/ajpgi.00440.2015, PMID: 27012776

[81] Kratsman N Getselter D Elliott E. Sodium butyrate attenuates social behavior deficits and modifies the transcription of inhibitory/excitatory genes in the frontal cortex of an autism model. Neuropharmacology. (2016) 102:136–45. doi: 10.1016/j.neuropharm.2015.11.003, PMID: 26577018

[82] PubMed. Rescue of maternal immune activation-induced behavioral abnormalities in adult mouse offspring by pathogen-activated maternal Treg cells. https://pubmed.ncbi.nlm.nih.gov/33859437/ (2023). (Accessed 5 May 2023). 10.1038/s41593-021-00837-1 33859437

[83] Si Z-Z Zou C-J Mei X Li X-F Luo H Shen Y. Targeting neuroinflammation in Alzheimer’s disease: from mechanisms to clinical applications. Neural Regen Res. (2022) 18:708–15. doi: 10.4103/1673-5374.353484 PMC9700083 36204826

[84] Breijyeh Z Karaman R. Comprehensive review on Alzheimer’s disease: causes and treatment. Molecules. (2020) 25:5789. doi: 10.3390/molecules25245789, PMID: 33302541 PMC7764106

[85] Jorfi M Maaser-Hecker A Tanzi RE. The neuroimmune axis of Alzheimer’s disease. Genome Med. (2023) 15:6. doi: 10.1186/s13073-023-01155-w 36703235 PMC9878767

[86] Hussain B Fang C Chang J. Blood-brain barrier breakdown: An emerging biomarker of cognitive impairment in Normal aging and dementia. Front Neurosci. (2021) 15:90. doi: 10.3389/fnins.2021.688090, PMID: 34489623 PMC8418300

[87] Zhang J Ke K-F Liu Z Qiu Y-H Peng Y-P. Th17 cell-mediated neuroinflammation is involved in neurodegeneration of aβ1-42-induced Alzheimer’s disease model rats. PLoS One. (2013) 8:e75786. doi: 10.1371/journal.pone.0075786, PMID: 24124514 PMC3790825

[88] Gautam AS Pulivarthi CB Singh RK. Proinflammatory IL-17 levels in serum/cerebrospinal fluid of patients with neurodegenerative diseases: a meta-analysis study. Naunyn Schmiedeberg's Arch Pharmacol. (2023) 396:577–88. doi: 10.1007/s00210-022-02357-6, PMID: 36504126

[89] Chen J-M Jiang G-X Li Q-W Zhou Z-M Cheng Q. Increased serum levels of interleukin-18, −23 and −17 in Chinese patients with Alzheimer’s disease. Dement Geriatr Cogn Disord. (2014) 38:321–9. doi: 10.1159/000360606, PMID: 25138786

[90] Cristiano C Volpicelli F Lippiello P Buono B Raucci F Piccolo M. Neutralization of IL-17 rescues amyloid-β-induced neuroinflammation and memory impairment. Br J Pharmacol. (2019) 176:3544–57. doi: 10.1111/bph.14586, PMID: 30673121 PMC6715610

[91] Ye X Chen J Pan J Wu Q Wang Y Lu M. Interleukin-17 promotes the infiltration of CD8+ T cells into the brain in a mouse model for Alzheimer’s disease. Immunol Investig. (2023) 52:135–53. doi: 10.1080/08820139.2022.2136525, PMID: 36394561

[92] Pirker-Kees A Schmied C Dal-Bianco P. T-cells show increased production of cytokines and activation markers in Alzheimer’s disease. Brain Disord Ther. (2013) 3:1–4. doi: 10.4172/2168-975X.1000112

[93] Oberstein TJ Taha L Spitzer P Hellstern J Herrmann M Kornhuber J. Imbalance of circulating Th17 and regulatory T cells in Alzheimer’s disease: a case control study. Front Immunol. (2018) 9:1213. doi: 10.3389/fimmu.2018.01213 29915582 PMC5994416

[94] Dansokho C Ait Ahmed D Aid S Toly-Ndour C Chaigneau T Calle V. Regulatory T cells delay disease progression in Alzheimer-like pathology. Brain. (2016) 139:1237–51. doi: 10.1093/brain/awv408, PMID: 26912648

[95] Baruch K Rosenzweig N Kertser A Deczkowska A Sharif AM Spinrad A. Breaking immune tolerance by targeting Foxp3(+) regulatory T cells mitigates Alzheimer’s disease pathology. Nat Commun. (2015) 6:7967. doi: 10.1038/ncomms8967 26284939 PMC4557123

[96] Sun L Ju T Wang T Zhang L Ding F Zhang Y. Decreased Netrin-1 and correlated Th17/Tregs balance disorder in Aβ1-42 induced Alzheimer’s disease model rats. Front Aging Neurosci. (2019) 11:124. doi: 10.3389/fnagi.2019.00124 31191297 PMC6548067

[97] Brigas HC Ribeiro M Coelho JE Gomes R Gomez-Murcia V Carvalho K. IL-17 triggers the onset of cognitive and synaptic deficits in early stages of Alzheimer’s disease. Cell Rep. (2021) 36:109574. doi: 10.1016/j.celrep.2021.109574, PMID: 34469732

[98] Zenaro E Pietronigro E Della Bianca V Piacentino G Marongiu L Budui S. Neutrophils promote Alzheimer’s disease-like pathology and cognitive decline via LFA-1 integrin. Nat Med. (2015) 21:880–6. doi: 10.1038/nm.3913, PMID: 26214837

[99] Sun Y Zhang H Zhang X Wang W Chen Y Cai Z. Promotion of astrocyte-neuron glutamate-glutamine shuttle by SCFA contributes to the alleviation of Alzheimer’s disease. Redox Biol. (2023) 62:102690. doi: 10.1016/j.redox.2023.102690, PMID: 37018970 PMC10122027

[100] Wang Y An Y Ma W Yu H Lu Y Zhang X. 27-hydroxycholesterol contributes to cognitive deficits in APP/PS1 transgenic mice through microbiota dysbiosis and intestinal barrier dysfunction. J Neuroinflammation. (2020) 17:199. doi: 10.1186/s12974-020-01873-7 32593306 PMC7321549

[101] Rosell-Cardona C Griñan-Ferré C Pérez-Bosque A Polo J Pallàs M Amat C. Dietary spray-dried porcine plasma reduces neuropathological Alzheimer’s disease hallmarks in SAMP8 mice. Nutrients. (2021) 13:2369. doi: 10.3390/nu13072369, PMID: 34836320 PMC8625036

[102] Willinger Y Turgeman G. Neuroprotective activity of Sitagliptin via reduction of Neuroinflammation beyond the incretin effect: focus on Alzheimer’s disease. Cells. (2022) 11:343. doi: 10.3390/cells11030343, PMID: 30186862 PMC6116461

[103] Milligan TA. Epilepsy: a clinical overview. Am J Med. (2021) 134:840–7. doi: 10.1016/j.amjmed.2021.01.038, PMID: 33775643

[104] Ding D Zhou D Sander JW Wang W Li S Hong Z. Epilepsy in China: major progress in the past two decades. Lancet Neurol. (2021) 20:316–26. doi: 10.1016/S1474-4422(21)00023-5, PMID: 33743240

[105] An J Li H Xia D Xu B Wang J Qiu H. The role of interleukin-17 in epilepsy. Epilepsy Res. (2022) 186:107001. doi: 10.1016/j.eplepsyres.2022.107001, PMID: 35994860

[106] Vieira ÉLM de Oliveira GNM Lessa JMK Gonçalves AP Oliveira ACP Bauer ME. Peripheral leukocyte profile in people with temporal lobe epilepsy reflects the associated proinflammatory state. Brain Behav Immun. (2016) 53:123–30. doi: 10.1016/j.bbi.2015.11.016, PMID: 26640228

[107] Burfeind KG Kashama J-MK Bora BK Murchison CF Ramos-Crawford AL Nseka MT. Baseline characterization of epilepsy in an onchocerciasis endemic area of the Democratic Republic of Congo. Brain Res Bull. (2019) 145:45–52. doi: 10.1016/j.brainresbull.2018.11.009, PMID: 30468846 PMC6377286

[108] Soltani Khaboushan A Yazdanpanah N Rezaei N. Neuroinflammation and Proinflammatory cytokines in Epileptogenesis. Mol Neurobiol. (2022) 59:1724–43. doi: 10.1007/s12035-022-02725-6, PMID: 35015252

[109] de Vries EE van den Munckhof B Braun KPJ van Royen-Kerkhof A de Jager W Jansen FE. Inflammatory mediators in human epilepsy: a systematic review and meta-analysis. Neurosci Biobehav Rev. (2016) 63:177–90. doi: 10.1016/j.neubiorev.2016.02.007, PMID: 26877106

[110] Xu D Robinson AP Ishii T Duncan DS Alden TD Goings GE. Peripherally derived T regulatory and γδ T cells have opposing roles in the pathogenesis of intractable pediatric epilepsy. J Exp Med. (2018) 215:1169–86. doi: 10.1084/jem.20171285, PMID: 29487082 PMC5881465

[111] Kumar P Shih DCW Lim A Paleja B Ling S Li Yun L. Proinflammatory IL-17 pathways dominate the architecture of the immunome in pediatric refractory epilepsy. JCI Insight. (2019) 4:e126337. doi: 10.1172/jci.insight.126337, PMID: 30912766 PMC6538358

[112] He J-J Sun F-J Wang Y Luo X-Q Lei P Zhou J. Increased expression of interleukin 17 in the cortex and hippocampus from patients with mesial temporal lobe epilepsy. J Neuroimmunol. (2016) 298:153–9. doi: 10.1016/j.jneuroim.2016.07.017, PMID: 27609289

[113] He J-J Wu K-F Li S Shu H-F Zhang C-Q Liu S-Y. Expression of the interleukin 17 in cortical tubers of the tuberous sclerosis complex. J Neuroimmunol. (2013) 262:85–91. doi: 10.1016/j.jneuroim.2013.05.007, PMID: 23906968

[114] He J-J Li S Shu H-F Yu S-X Liu S-Y Yin Q. The interleukin 17 system in cortical lesions in focal cortical dysplasias. J Neuropathol Exp Neurol. (2013) 72:152–63. doi: 10.1097/NEN.0b013e318281262e, PMID: 23334598

[115] Luo H Liu H-Z Zhang W-W Matsuda M Lv N Chen G. Interleukin-17 regulates neuron-glial communications, synaptic transmission, and neuropathic pain after chemotherapy. Cell Rep. (2019) 29:2384–2397.e5. doi: 10.1016/j.celrep.2019.10.085, PMID: 31747607

[116] Mazdeh M Omrani MD Sayad A Komaki A Arsang-Jang S Taheri M. Expression analysis of cytokine coding genes in epileptic patients. Cytokine. (2018) 110:284–7. doi: 10.1016/j.cyto.2018.01.017, PMID: 29396051

[117] Ouédraogo O Rébillard R-M Jamann H Mamane VH Clénet M-L Daigneault A. Increased frequency of proinflammatory CD4 T cells and pathological levels of serum neurofilament light chain in adult drug-resistant epilepsy. Epilepsia. (2021) 62:176–89. doi: 10.1111/epi.16742, PMID: 33140401

[118] Wei J Liu H Liu Z Jiang X Wang W. The temporal and spatial changes of Th17, Tregs, and related cytokines in epilepsy lesions. Appl Bionics Biomech. (2022) 2022:7871302. doi: 10.1155/2022/7871302, PMID: 35528532 PMC9071937

[119] Ni F-F Li C-R Liao J-X Wang G-B Lin S-F Xia Y. The effects of ketogenic diet on the Th17/Treg cells imbalance in patients with intractable childhood epilepsy. Seizure. (2016) 38:17–22. doi: 10.1016/j.seizure.2016.03.006, PMID: 27061881

[120] Moynes DM Vanner SJ Lomax AE. Participation of interleukin 17A in neuroimmune interactions. Brain Behav Immun. (2014) 41:1–9. doi: 10.1016/j.bbi.2014.03.004, PMID: 24642072

[121] Lang JD Olmes DG Proske M Hagge M Dogan Onugoren M Rothhammer V. Pre- and postictal changes in the innate immune system: cause or effect? Eur Neurol. (2021) 84:380–8. doi: 10.1159/000516556 34139710

[122] Yue Q Cai M Xiao B Zhan Q Zeng C. The microbiota–gut–brain Axis and epilepsy. Cell Mol Neurobiol. (2022) 42:439–53. doi: 10.1007/s10571-021-01130-2, PMID: 34279746 PMC11441249

[123] Xie G Zhou Q Qiu C-Z Dai W-K Wang H-P Li Y-H. Ketogenic diet poses a significant effect on imbalanced gut microbiota in infants with refractory epilepsy. World J Gastroenterol. (2017) 23:6164–71. doi: 10.3748/wjg.v23.i33.6164, PMID: 28970732 PMC5597508

[124] Kilinc E Ankarali S Ayhan D Ankarali H Torun IE Cetinkaya A. Protective effects of long-term probiotic mixture supplementation against pentylenetetrazole-induced seizures, inflammation and oxidative stress in rats. J Nutr Biochem. (2021) 98:108830. doi: 10.1016/j.jnutbio.2021.108830, PMID: 34333116

[125] Wu J Zhang Y Yang H Rao Y Miao J Lu X. Intestinal microbiota as an alternative therapeutic target for epilepsy. Can J Infect Dis Med Microbiol. (2016) 2016:9032809. doi: 10.1155/2016/9032809, PMID: 27882059 PMC5108868

[126] Marzocco S Fazeli G Di Micco L Autore G Adesso S Dal Piaz F. Supplementation of short-chain fatty acid, sodium propionate, in patients on maintenance hemodialysis: beneficial effects on inflammatory parameters and gut-derived uremic toxins, a pilot study (PLAN study). J Clin Med. (2018) 7:315. doi: 10.3390/jcm7100315, PMID: 30274359 PMC6210519

[127] De Caro C Leo A Nesci V Ghelardini C di Cesare ML Striano P. Intestinal inflammation increases convulsant activity and reduces antiepileptic drug efficacy in a mouse model of epilepsy. Sci Rep. (2019) 9:13983. doi: 10.1038/s41598-019-50542-0 31562378 PMC6764994

[128] Himmerich H Bartsch S Hamer H Mergl R Schönherr J Petersein C. Modulation of cytokine production by drugs with antiepileptic or mood stabilizer properties in anti-CD3- and anti-Cd40-stimulated blood in vitro. Oxidative Med Cell Longev. (2014) 2014:806162. doi: 10.1155/2014/806162, PMID: 24757498 PMC3976773

[129] Hao Y Ge H Sun M Gao Y. Selecting an appropriate animal model of depression. Int J Mol Sci. (2019) 20:4827. doi: 10.3390/ijms20194827, PMID: 31569393 PMC6801385

[130] Shorey S Ng ED Wong CHJ. Global prevalence of depression and elevated depressive symptoms among adolescents: a systematic review and meta-analysis. Br J Clin Psychol. (2022) 61:287–305. doi: 10.1111/bjc.12333, PMID: 34569066

[131] Kiecolt-Glaser JK Derry HM Fagundes CP. Inflammation: depression fans the flames and feasts on the heat. Am J Psychiatry. (2015) 172:1075–91. doi: 10.1176/appi.ajp.2015.15020152, PMID: 26357876 PMC6511978

[132] Mao L Ren X Wang X Tian F. Associations between autoimmunity and depression: serum IL-6 and IL-17 have directly impact on the HAMD scores in patients with first-episode depressive disorder. J Immunol Res. (2022) 2022:6724881. doi: 10.1155/2022/6724881, PMID: 35615531 PMC9126704

[133] Chen Y Jiang T Chen P Ouyang J Xu G Zeng Z. Emerging tendency towards autoimmune process in major depressive patients: a novel insight from Th17 cells. Psychiatry Res. (2011) 188:224–30. doi: 10.1016/j.psychres.2010.10.029, PMID: 21129782

[134] Beurel E Lowell JA. Th17 cells in depression. Brain Behav Immun. (2018) 69:28–34. doi: 10.1016/j.bbi.2017.08.001, PMID: 28779999 PMC5797502

[135] Gu M Li Y Tang H Zhang C Li W Zhang Y. Endogenous omega (n)-3 fatty acids in Fat-1 mice attenuated depression-like behavior, imbalance between microglial M1 and M2 phenotypes, and dysfunction of Neurotrophins induced by lipopolysaccharide administration. Nutrients. (2018) 10:1351. doi: 10.3390/nu10101351, PMID: 30248907 PMC6213921

[136] Nadeem A Ahmad SF Al-Harbi NO Fardan AS El-Sherbeeny AM Ibrahim KE. IL-17A causes depression-like symptoms via NFκB and p38MAPK signaling pathways in mice: implications for psoriasis associated depression. Cytokine. (2017) 97:14–24. doi: 10.1016/j.cyto.2017.05.018, PMID: 28570931

[137] Beurel E Lowell J Jope R. Distinct characteristics of hippocampal pathogenic TH17 cells in a mouse model of depression. Brain Behav Immun. (2018) 73:180–91. doi: 10.1016/j.bbi.2018.04.012, PMID: 29698707 PMC6287768

[138] Phillips JGP. The treatment of melancholia by the lactic acid Bacillus. J Ment Sci. (1910) 56:422–30. doi: 10.1192/bjp.56.234.422

[139] Zhu X Sakamoto S Ishii C Smith MD Ito K Obayashi M. Dectin-1 signaling on colonic γδ T cells promotes psychosocial stress responses. Nat Immunol. (2023) 24:625–36. doi: 10.1038/s41590-023-01447-8, PMID: 36941398 PMC10704545

[140] Bravo JA Forsythe P Chew MV Escaravage E Savignac HM Dinan TG. Ingestion of Lactobacillus strain regulates emotional behavior and central GABA receptor expression in a mouse via the vagus nerve. Proc Natl Acad Sci U S A. (2011) 108:16050–5. doi: 10.1073/pnas.1102999108, PMID: 21876150 PMC3179073

[141] Leonardi I Gao IH Lin W-Y Allen M Li XV Fiers WD. Mucosal fungi promote gut barrier function and social behavior via type 17 immunity. Cells. (2022) 185:831–846.e14. doi: 10.1016/j.cell.2022.01.017, PMID: 35176228 PMC8897247

[142] Pistorio E Luca M Luca A Messina V Calandra C. Autonomic nervous system and lipid metabolism: findings in anxious-depressive spectrum and eating disorders. Lipids Health Dis. (2011) 10:192. doi: 10.1186/1476-511X-10-192 22034981 PMC3215932

[143] Zou L Tian Y Wang Y Chen D Lu X Zeng Z. High-cholesterol diet promotes depression- and anxiety-like behaviors in mice by impact gut microbe and neuroinflammation. J Affect Disord. (2023) 327:425–38. doi: 10.1016/j.jad.2023.01.122, PMID: 36738999

[144] Do Sacramento PM Sales M Kasahara De MT Monteiro C Oyamada H ASO D. Major depression favors the expansion of Th17-like cells and decrease the proportion of CD39+Treg cell subsets in response to myelin antigen in multiple sclerosis patients. Cell Mol Life Sci. (2022) 79:298. doi: 10.1007/s00018-022-04315-0, PMID: 35585332 PMC11073410

[145] Sun M Ma N He T Johnston LJ Ma X. Tryptophan (Trp) modulates gut homeostasis via aryl hydrocarbon receptor (AhR). Crit Rev Food Sci Nutr. (2020) 60:1760–8. doi: 10.1080/10408398.2019.1598334, PMID: 30924357

[146] Tsai Y-L Lin T-L Chang C-J Wu T-R Lai W-F Lu C-C. Probiotics, prebiotics and amelioration of diseases. J Biomed Sci. (2019) 26:3. doi: 10.1186/s12929-018-0493-6, PMID: 30609922 PMC6320572

[147] Puricelli C Rolla R Gigliotti L Boggio E Beltrami E Dianzani U. The gut-brain-immune Axis in autism Spectrum disorders: a state-of-art report. Front Psych. (2022) 12:755171. doi: 10.3389/fpsyt.2021.755171, PMID: 35185631 PMC8850385

[148] Kebir H Kreymborg K Ifergan I Dodelet-Devillers A Cayrol R Bernard M. Human TH17 lymphocytes promote blood-brain barrier disruption and central nervous system inflammation. Nat Med. (2007) 13:1173–5. doi: 10.1038/nm1651, PMID: 17828272 PMC5114125

[149] Bartsch T Wulff P. The hippocampus in aging and disease: from plasticity to vulnerability. Neuroscience. (2015) 309:1–16. doi: 10.1016/j.neuroscience.2015.07.084, PMID: 26241337

[150] Patel DC Tewari BP Chaunsali L Sontheimer H. Neuron–glia interactions in the pathophysiology of epilepsy. Nat Rev Neurosci. (2019) 20:282–97. doi: 10.1038/s41583-019-0126-4, PMID: 30792501 PMC8558781

[151] Allen NJ Lyons DA. Glia as architects of central nervous system formation and function. Science. (2018) 362:181–5. doi: 10.1126/science.aat0473, PMID: 30309945 PMC6292669

